# The overweight and obesity transition from the wealthy to the poor in low- and middle-income countries: A survey of household data from 103 countries

**DOI:** 10.1371/journal.pmed.1002968

**Published:** 2019-11-27

**Authors:** Tara Templin, Tiago Cravo Oliveira Hashiguchi, Blake Thomson, Joseph Dieleman, Eran Bendavid

**Affiliations:** 1 Department of Health Research and Policy, Stanford University School of Medicine, Stanford, California, United States of America; 2 Organisation for Economic Co-operation and Development ELS/HD, Paris, France; 3 Nuffield Department of Population Health, University of Oxford, Oxford, United Kingdom; 4 Institute for Health Metrics and Evaluation, University of Washington, Seattle, Washington, United States of America; 5 Center for Population Health Sciences, Division of Primary Care and Population Health, Department of Medicine, Stanford University, Stanford, California, United States of America; Carolina Population Center, UNITED STATES

## Abstract

**Background:**

In high-income countries, obesity prevalence (body mass index greater than or equal to 30 kg/m^2^) is highest among the poor, while overweight (body mass index greater than or equal to 25 kg/m^2^) is prevalent across all wealth groups. In contrast, in low-income countries, the prevalence of overweight and obesity is higher among wealthier individuals than among poorer individuals. We characterize the transition of overweight and obesity from wealthier to poorer populations as countries develop, and project the burden of overweight and obesity among the poor for 103 countries.

**Methods and findings:**

Our sample used 182 Demographic and Health Surveys and World Health Surveys (*n* = 2.24 million respondents) from 1995 to 2016. We created a standard wealth index using household assets common among all surveys and linked national wealth by country and year identifiers. We then estimated the changing probability of overweight and obesity across every wealth decile as countries’ per capita gross domestic product (GDP) rises using logistic and linear fixed-effect regression models. We found that obesity rates among the wealthiest decile were relatively stable with increasing national wealth, and the changing gradient was largely due to increasing obesity prevalence among poorer populations (3.5% [95% uncertainty interval: 0.0%–8.3%] to 14.3% [9.7%–19.0%]). Overweight prevalence among the richest (45.0% [35.6%–54.4%]) and the poorest (45.5% [35.9%–55.0%]) were roughly equal in high-income settings. At $8,000 GDP per capita, the adjusted probability of being obese was no longer highest in the richest decile, and the same was true of overweight at $10,000. Above $25,000, individuals in the richest decile were less likely than those in the poorest decile to be obese, and the same was true of overweight at $50,000. We then projected overweight and obesity rates by wealth decile to 2040 for all countries to quantify the expected rise in prevalence in the relatively poor. Our projections indicated that, if past trends continued, the number of people who are poor and overweight will increase in our study countries by a median 84.4% (range 3.54%–383.4%), most prominently in low-income countries. The main limitations of this study included the inclusion of cross-sectional, self-reported data, possible reverse causality of overweight and obesity on wealth, and the lack of physical activity and food price data.

**Conclusions:**

Our findings indicate that as countries develop economically, overweight prevalence increased substantially among the poorest and stayed mostly unchanged among the wealthiest. The relative poor in upper- and lower-middle income countries may have the greatest burden, indicating important planning and targeting needs for national health programs.

## Abstract 2

Tara Templin and colleagues project the burden of obesity in 103 countries.

## Author summary

**Why was this study done?:**

- Obesity prevalence has been rising in every country in the world since 1975 and contributes to an increasing proportion of noncommunicable disease risk and burden.
- Within countries, obesity prevalence is highest among wealthier population strata in poorer countries, but the burden of obesity shifts to poorer population strata as national wealth increases.
- While the flipped wealth gradients of obesity in poor and rich countries are documented, no research shows how this shift happens or where it occurs along the range of economic development.
- This work was motivated by a desire to inform policy makers of when and how this reversal is likely to occur, because obesity among the poor has different implications for public health policy than obesity among the wealthy.

**What did the researchers do and find?:**

- We collated 182 Demographic and Health Surveys and World Health Surveys [*n* = 2.24 million respondents] from 103 countries with information on respondent height, weight, personal wealth, and country wealth.
- We estimated the relationship of personal wealth with overweight and obesity, and examined how that relationship varies with country wealth.
- At a gross domestic product (GDP) per capita of $8,000, the prevalence of obesity is no longer the highest among those in the top wealth decile ($10,000 for overweight), and at $30,000, obesity prevalence among those in the poorest decile is higher than among the wealthiest ($50,000 for overweight). The transition is driven by increasing obesity among the poor without appreciable decreasing obesity among the wealthy.
- Above $10,000 GDP per capita, the relationship between overweight and personal wealth starts to change as overweight prevalence among those in the lower deciles of wealth increases.
- We estimate that 70.2% (64.1%–76.4%) of countries will begin or have undergone this shift by 2040, and the number of people who are both obese and poor (in the lowest quintile) in our study countries will increase by 17.3 million people (15.3–19.6).

**What do these findings mean?:**

- Distinct points along the obesity transition may represent a particularly effective time for policy makers to implement obesity interventions.
- At a GDP per capita of $8,000, the burden of paying for the costs of obesity may start to increase, as the prevalence of obesity is no longer uniquely concentrated among the wealthy.
- People in the richest decile may not be affected by the same economic mechanisms that increase obesity in the poor, as obesity prevalence of the wealthy within each country does not change substantially with economic development.

## Introduction

Since 1975, the worldwide prevalence of overweight (body mass index (BMI) greater than or equal to 25 kg/m^2^) among adults increased from 21.5% to 38.9% in 2016 [1]. Over this period, no country has experienced a decline in the prevalence of overweight or obesity (BMI greater than or equal to 30 kg/m^2^) [2]. In 2000, the World Health Organization declared obesity a pandemic and 12 years later issued a global action plan to combat its rise [3,4]. The rapid rise in overweight and the inefficacy of population-level control measures are thought to be important drivers of the rising disease burden and mortality from cardiovascular disease, cancer, and diabetes.

Unlike many health conditions, such as diarrheal illnesses and respiratory infections [5], in which the poor consistently carry a disproportionate share of the health burden irrespective of national aggregate wealth, overweight and obesity do not display a consistent wealth gradient across different levels of economic development. In most low- and middle-income countries, the prevalence of overweight and obesity is higher among wealthier individuals than among poorer. However, as national economic output increases, the burden of overweight and obesity shifts to populations with lower personal wealth [6–8]. The question of at what point along the spectrum of national economic development the burden of overweight and obesity shifts to the poor is thus an important one, which has received limited attention.

This shift in overweight and obesity burden from the rich to the poor is an important consequence of economic development, yet it receives little policy attention [9]. Industrialization, urbanization, and the nutritional transition are commonly posited as drivers of overweight and obesity with increasing country wealth [6–8,10–13]. These studies suggest that food consumption patterns change differentially in different wealth strata as countries become wealthier. While there is no singular reason for these trends, increased supply of processed food (greater integration into the global economy brings more processed foods [11,13]), price differences (per calorie, processed foods are less costly than non-processed foods [14]), and personal wealth changes could all be associated with changing patterns in food consumption. Along with worse healthcare and worse health outcomes, more overweight among the relatively poor is a major issue confronting public health programs.

This shift in burden has three direct implications. First, characterizing the overweight and obesity burden shift is fundamental to understanding its drivers, including the economic and nutritional drivers. Second, the measurement of burden inequities can inform understanding of the changing distribution of cardiometabolic diseases. Third, the mix of available and effective interventions to prevent overweight and treat overweight-related diseases may vary based on the personal wealth of those in need, indicating important planning and targeting needs for national health programs.

In this analysis, we aim to identify a wealth-overweight transition zone, which is a term we will use to denote the range of gross domestic product (GDP) per capita where overweight burden shifts within countries from the wealthy to the poor. This work contributes to the extant literature by characterizing the wealth-overweight transition zone, with potential implications for mitigation strategies. The analysis has three aims: to (1) characterize overweight and obesity wealth gradients as countries develop economically; (2) identify the range of GDP per capita where the transition occurs; and (3) project overweight and obesity burden transitions to 2040.

## Methods

### Data sources

We extracted data from two series of surveys: the Demographic and Health Surveys and the World Health Surveys [15,16]. These are household, cross-sectional surveys that employ a multistage sampling design. The Demographic and Health Surveys collect anthropometrically measured height and weight from all respondents, as well as a series of asset indicators meant to capture personal wealth. The World Health Surveys collect mostly self-reported height and weight measurements, along with many asset indicators. From these sources, we compiled 182 surveys, representing 103 countries across different time points from 1995 to 2015. A total of 2.24 million survey respondents aged 15–49 were included in the sample. Detailed information concerning the surveys can be found in Tables A–F and Figs A–B in S1 Appendix.

### Measurements of overweight and obesity

We categorized adults aged 18 and over with a BMI of 30 kg/m^2^ or greater as obese and those with a BMI of 25 kg/m^2^ or greater as overweight. We will refer to overweight, inclusive of obesity, as “overweight” throughout the paper. For survey respondents aged 15–17, classification was made according to the International Obesity Task Force's growth curve [17]. Adults with missing or implausible BMI measurements (less than 12 or greater than 60) were excluded (more information is available in S1 Appendix Section 2.6). Height and weight data in the World Health Surveys are self-reported, and some studies have shown bias in self-reporting of height and weight [18]. We conducted sensitivity analyses to test for systematic differences between overlapping Demographic and Health Surveys and World Health Surveys and found no significant bias (Fig C and Tables H, J, L in S1 Appendix).

### National income

We used GDP per capita collected from the Institute for Health Metrics and Evaluation as a measure of national income in constant 2017 purchasing power parity adjusted dollars [19]. This data source also projects GDP for all countries in our study up to 2040, which we use to project overweight and obesity distribution trends. The World Health Surveys include countries spanning from the Democratic Republic of the Congo ($1,000 per person per year) to Luxembourg ($112,000 per person per year). The Demographic and Health Surveys focus mostly on low- and middle-income countries, with Gabon at $18,000 having the highest GDP per capita in that sample and the Democratic Republic of the Congo ($1,000 per person per year) having the lowest. We linked GDP per capita to surveys by country and year identifiers.

### Individual wealth index

To capture personal wealth, we constructed a household-specific wealth index based on asset ownership. First, we collected survey-specific wealth ranks, meaning relative rank order of personal wealth within a country at the time of the survey based on asset inventories. We use the relative personal wealth rank because we can obtain it directly from each of our 103 surveys using a premade (Demographic and Health Surveys) or easily-derived (World Health Surveys) index from principal components analyses of wealth questionnaire responses. To calculate the first principal component, we use all available asset indicators for each country, and perform country-specific principal component analyses.

As a robustness check, we also calculate a second personal wealth index that is comparable across countries and years. In order to obtain this second wealth index, we perform principal components analysis on nine asset measures widely available in the Demographic and Health Surveys: water access, sanitation, floor material, electricity, refrigerator, motorcycle, car, phone, and rooms per person. The personal wealth index is the first principal component, which captures the maximal variance amongst these assets. We then use the index scores across the entire study population (across all surveys) to calculate wealth deciles.

### Statistical analysis

No prospective analysis plan was specified for this study. This work solely uses secondary, de-identified data and did not require ethics approval. As the study question concerns within-country changes, we started with a fixed-effects regression specification for both our wealth-overweight transition estimation and projections; additional sensitivity analyses and a meta-analytic technique are described below. This study is reported as per the Strengthening the Reporting of Observational Studies in Epidemiology (STROBE) guideline (S1 Checklist).

### Wealth-overweight transition estimation

We conducted multivariate regression analyses using the individual survey respondent as the unit of observation. The purpose of these models is to estimate how the wealth-overweight gradient changes with economic development. We do this by estimating the following regression equation, with a binomial outcome distribution and logit link function:

$${Overweight}_{ict}=\alpha +\beta ln({GDPpercapita}_{ct})+\sum_{w=1}^{10}\varphi_{w}{Wealth}_{i}^{w}+\sum_{w=1}^{10}\delta_{w}ln({GDPpercapita}_{ct})\times {Wealth}_{i}^{w}+\gamma {Age}_{ict}+\zeta {Female}_{ict}+\tau_{t}+\mu_{c}$$ (1)

where individuals are indexed by *i*, country by *c*, and year by *t*. The main coefficients of interest, *δ_w_*, represent the changing probability of overweight in individuals in each wealth decile, *w*, with increasing GDP per capita (logged). Individual controls include age (in years) and sex (indicator for female). We include country fixed effects, *μ_c_*, a series of binary indicators for each country, and year fixed effects, *τ_t_*, a series of binary indicators for each year. The country fixed effects remove all time-invariant differences between countries and allow us to examine the changing relationship of personal wealth with overweight within countries; that is, we estimate how overweight changes for a single wealth decile in a single country as that country’s GDP changes. Year fixed effects control for common time trends shared among all countries, in effect allowing us to estimate our relationship of interest after removing global trends in overweight. We estimate logistic models for the binary outcome variable of individual overweight and include findings with obesity and BMI as the dependent variables in Tables G–L in S1 Appendix. We also estimate the same model with country-specific age and sex trends in Fig E and wealth quintiles in Fig F in S1 Appendix. Finally, we also conduct a meta-regression analysis to estimate the relationship of GDP per capita to within-country effects of wealth on overweight, obesity, and BMI, available in Figs G–L and Tables N–S in S1 Appendix.

### Wealth-overweight transition projections

After estimating the basic shift in overweight and obesity trends between the poor and wealthy, we projected this shift to 2040. We project the overweight and obesity prevalence to 2040 of each sex and 5-year age groups, from age 15 to 49, using Eq 1 with a linear time trend. We use GDP per capita projections from the Institute for Health Metrics and Evaluation to estimate the effect of economic development to 2040 [19].

We incorporated variance in the GDP per capita series and parameter uncertainty from our regression model to quantify an uncertainty interval for our projections. After estimation of Eq 1, we took 1,000 draws from the multivariate normal distribution defined by the model parameter estimates and the variance-covariance matrix of the model. To create our predictions, those were coupled with 1,000 draws provided by the Institute for Health Metrics and Evaluation for their GDP per capita series. These draws incorporate model, data, and parameter uncertainty by using ensemble modeling techniques, drawing from the sub-model variance-covariance matrices, and adding a random walk of statistical noise to each forecast with variance based on the residual of the observed data for each sub-model. We weight the predicted age–sex-specific overweight and obesity rates by the United Nations World Population Prospects age–sex-specific population projections to aggregate overweight and obesity rates to the national level by personal wealth decile [20]. Thus, these projections also capture the effects of aging on overweight and obesity rates. In Table U in S1 Appendix, we report results for out-of-sample validation of these projections.

All analyses were conducted using Stata 14.1 and R 3.5.0.

## Results

**Fig 1:**
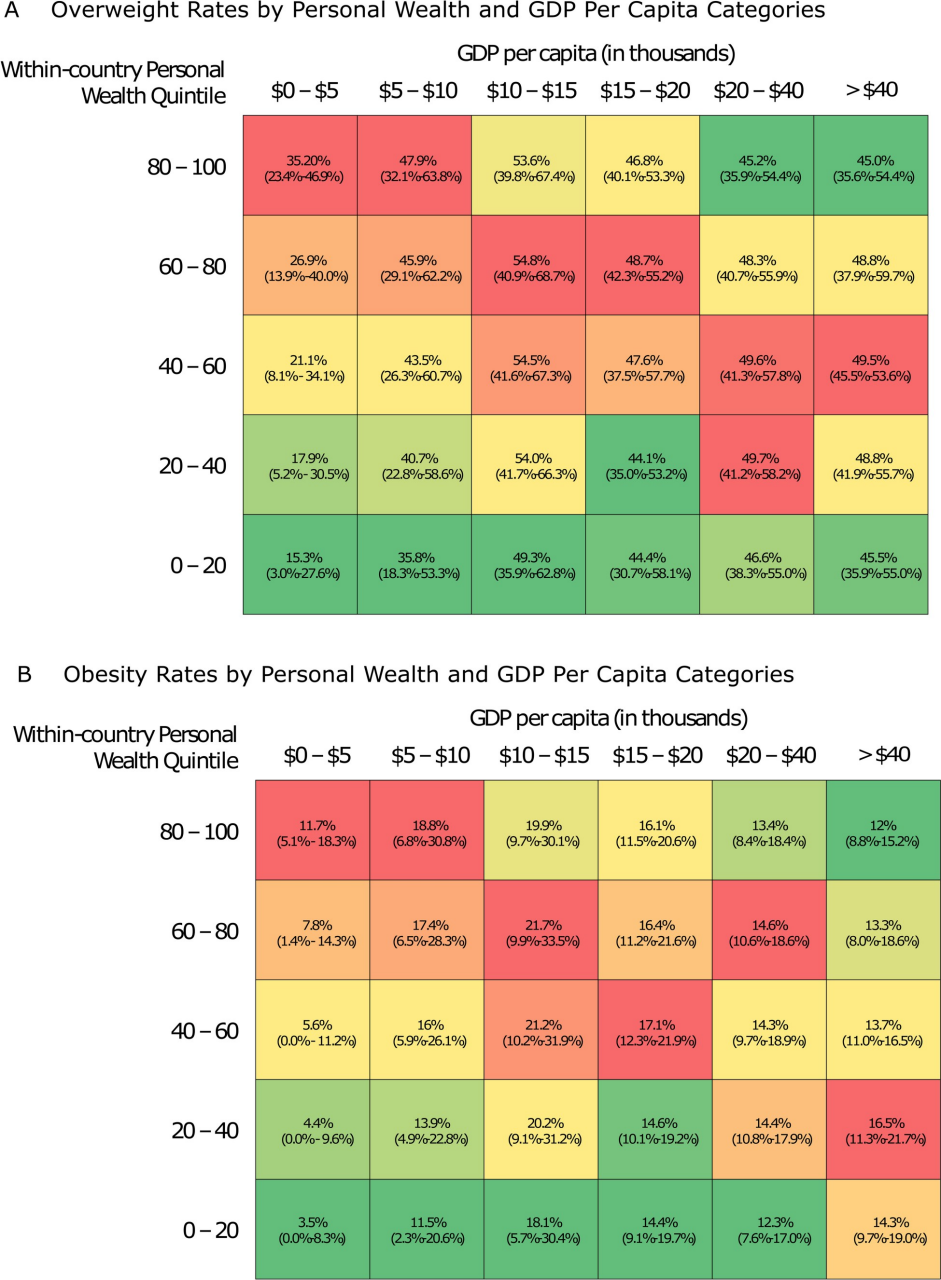
Overweight and obesity prevalence rates by economic development and within-survey wealth decile. Unadjusted overweight (1A) and obesity (1B) prevalence obtained directly from survey data, stratified by GDP per capita and within-survey personal wealth decile. The columns represent GDP per capita categories, and the rows represent deciles of within-country wealth. Within each GDP per capita category, deciles with the lowest prevalence are coded in green, and deciles with the highest prevalence are coded in red. All prevalence estimates were obtained using survey weights. GDP, gross domestic product.

**Fig 2:**
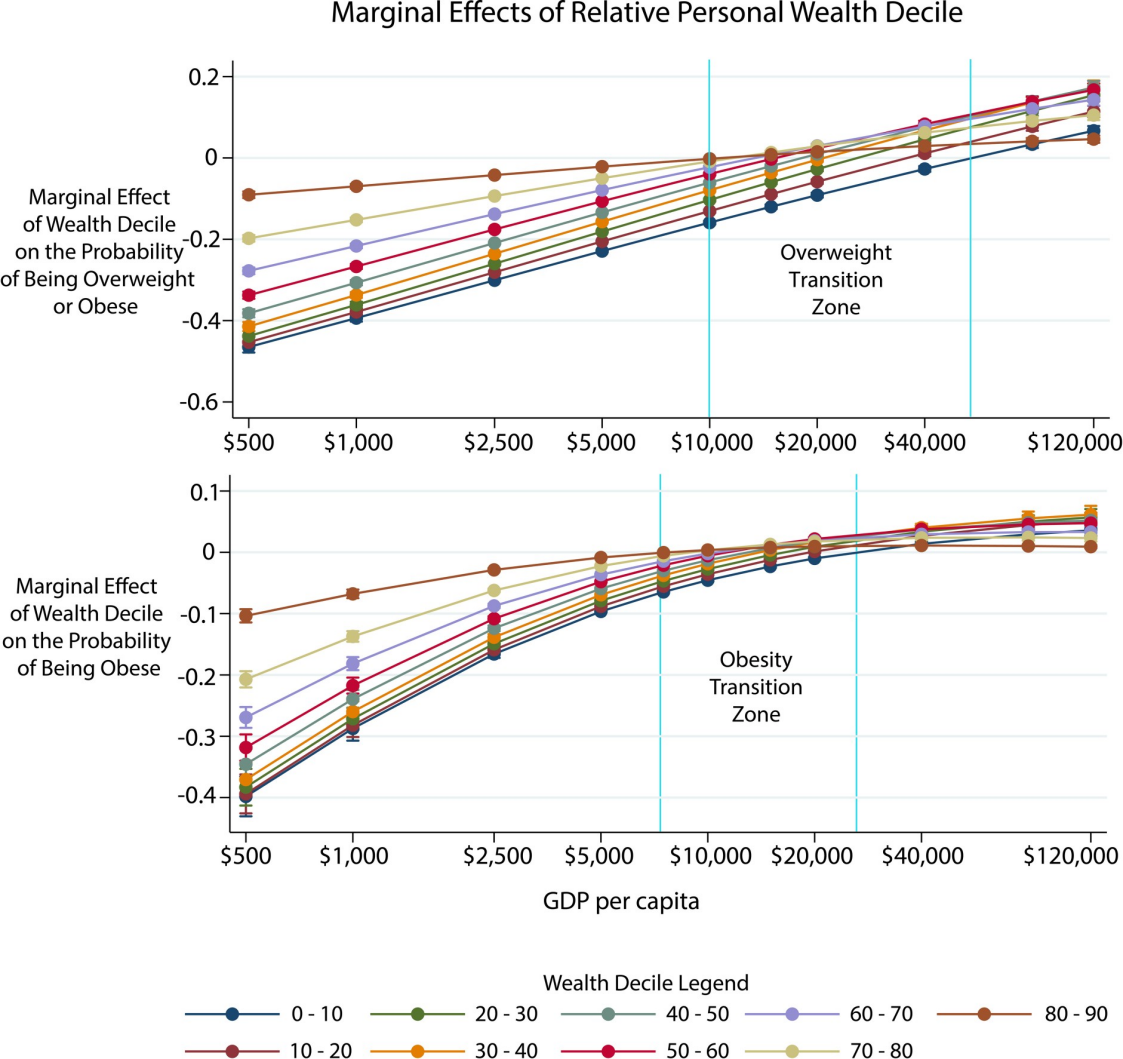
Differential effect of wealth on overweight and obesity by GDP per capita. Each point represents the probability of being overweight (2A) or obese (2B) relative to the richest decile (90th–100th percentile) at different GDP per capita cutoffs. Each error bar represents robust 95% confidence intervals. The lines are color coded by wealth decile. The wealth-overweight and wealth-obesity transition zones are denoted by the vertical lines. The first line marks where the richest decile was no longer the most likely to be overweight or obese. The second line marks where the richest decile was less likely than the poorest to be overweight or obese. We evaluate the change in the gradient based on where the other percentiles are statistically significantly greater than zero (which means the wealth group has a higher chance of obesity than the 90th–100th percentile personal wealth income group). GDP, gross domestic product.

We collected data from 182 nationally representative surveys with individual-level data for 2.24 million respondents. Fig 1 stratifies the overweight (1A) and obesity (1B) prevalence by level of economic development and ranked quintile of personal wealth. While Fig 2 adjusted for confounders, the shifting pattern was seen from the raw data. In the $0 to $5,000 category, the richest had an overweight prevalence of 35.2% (95% uncertainty interval: 23.4%–46.9%) and an obesity prevalence of 11.7% (5.1%–18.3%), while the poorest had an overweight prevalence of 15.3% (3.0%–27.6%) and an obesity prevalence of 3.5% (0.0%–8.3%). In the greater than $40,000 category, the richest had an obesity prevalence of 12.0% (8.8%–15.2%), while the poorest increased to an obesity prevalence of 14.3% (9.7%–19.0%). Overweight prevalence among the richest (45.0% [35.6%–54.4%]) and the poorest (45.5% [35.9%–55.0%]) was roughly equal.

Fig 2 reports the adjusted probability of being overweight (2A) and obese (2B) in each individual-level wealth decile (relative to the richest decile) with increasing GDP per capita. At a GDP per capita of $500, an individual in the lowest decile of personal wealth was 39.8% (36.6%–43.1%) less likely to be obese and 46.5% (45.1%–47.9%) less likely to be overweight compared with an individual in the richest decile (the reference case). At the GDP per capita of low-income countries, there was an increasing probability of overweight and obesity with personal wealth relative to individuals in the poorest decile. The wealth-overweight gradient began to shift at a GDP per capita around $10,000: (i) individuals in the richest decile no longer had the highest probability of obesity; and (ii) the relative probability of being obese for individuals in the poorest deciles had roughly doubled compared with $500 GDP per capita. This same shift happened in the obesity gradient at a GDP per capita of $8,000. Finally, around a GDP per capita of $50,000, individuals in the poorest deciles were more likely than the wealthiest decile to be overweight. This point in the wealth-obesity transition occurred at a GDP per capita of $25,000.

**Fig 3:**
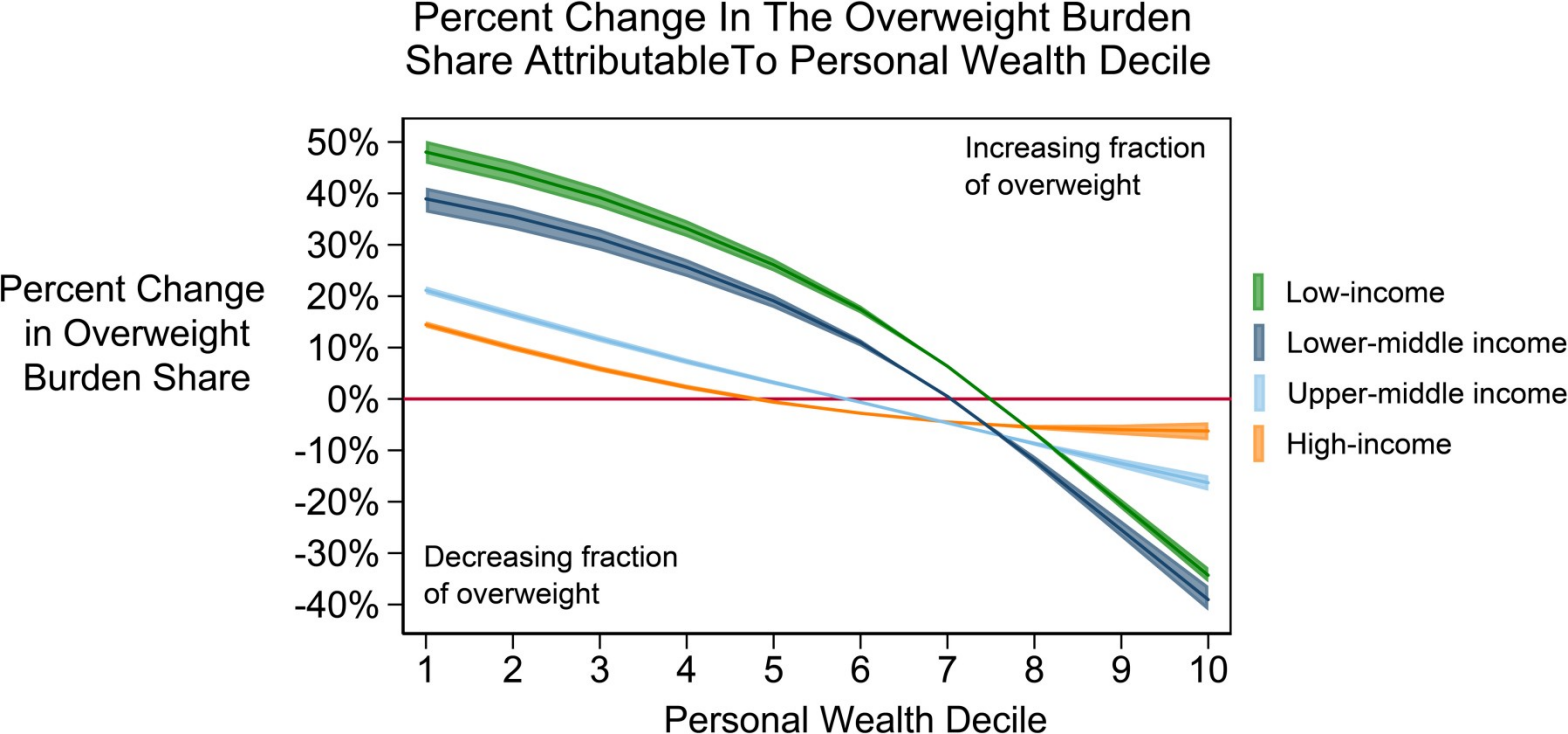
Change in the wealth distribution of the overweight population between 1995 and 2040. Each line displays World Bank Income group changes in overweight burden across a wealth decile. Wealth deciles, where 1 is the poorest and 10 is the richest, are displayed on the x-axis. The percent change in each wealth decile's overweight burden share from 1995 to 2040 is on the y-axis.

Fig 3 displays the projected relative change in the share of overweight by wealth decile between 1995 and 2040, grouped by World Bank income group. In low-income countries, the richest decile had a 33.6% (32.8%–35.7%) decline in the share of the overweight, while the poorest decile had a 47.0% (45.8%–50.2%) increase. The changes in the relative share of overweight were greatest in low- and lower-middle-income countries. In lower-middle-income countries, the share of overweight in the poorest decile was projected to increase 37.9% (36.3%–41.1%), while the share in the wealthiest decile is projected to decrease 38.4% (36.49%–41.2%). In high-income countries, overweight in the richest decile was expected to decrease 6.3% (4.6%–8.0%) and in the poorest decile to increase 14.1% (13.9%–15.1%).

**Fig 4:**
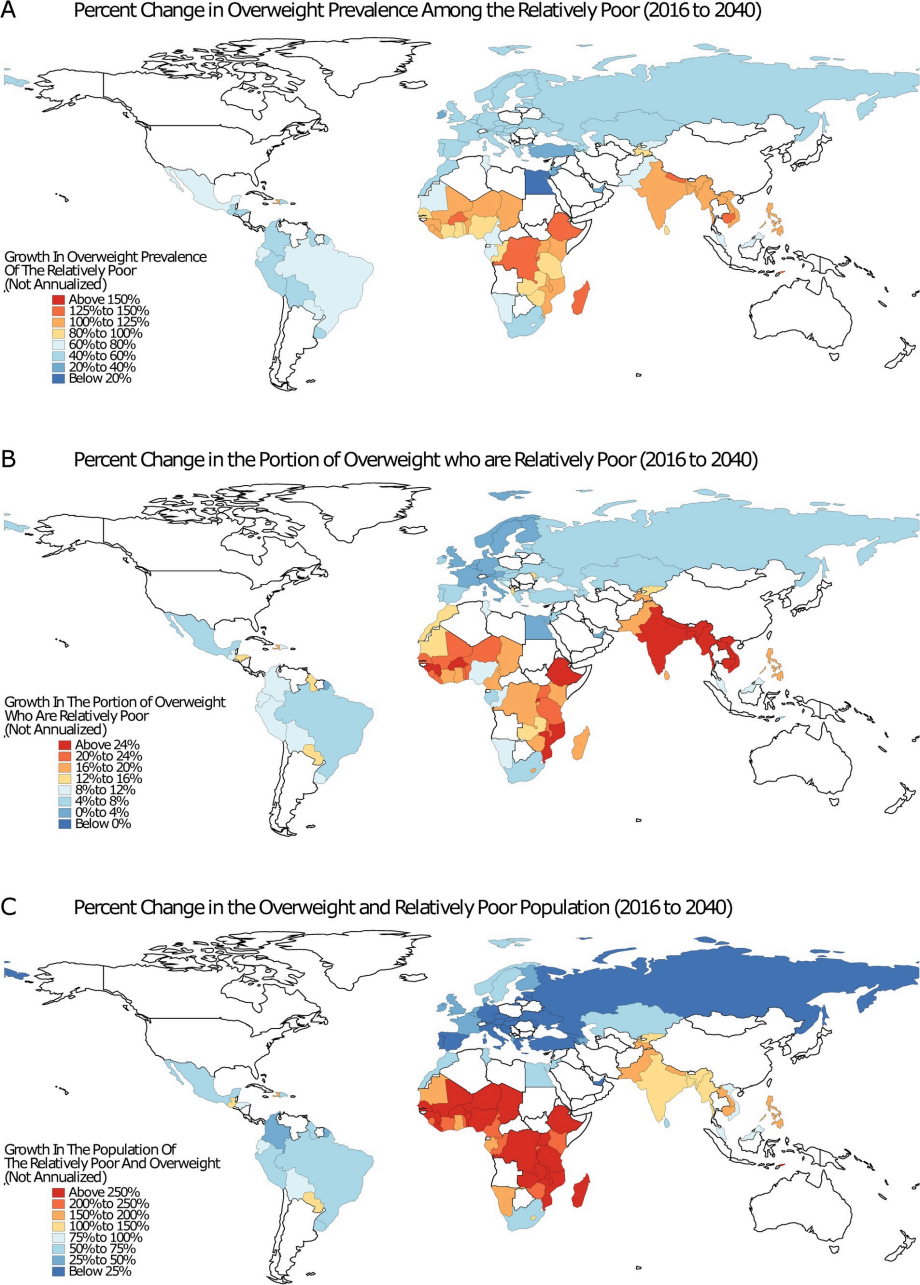
Overweight inequality projections, 2016 to 2040. Each map displays country-level projections in overweight prevalence inequality. Map A shows the percent change in overweight prevalence among the relatively poor, defined as individuals in the bottom quintile of the personal wealth distribution. Map B displays the percent change in the share of overweight individuals who are relatively poor. This differs from Map A by quantifying where the reversal of the wealth-overweight gradient will occur the fastest. Map C displays the percent change in the overweight and relatively poor population. *The base map was obtained from Natural Earth (https://naturalearthdata.com)*.

The maps in Fig 4 display the projected change in overweight prevalence, portion of overweight, and overweight population in the bottom quintile of personal wealth in each of our study countries (2016 to 2040). Fig 4A shows that the Democratic Republic of the Congo, Burkina Faso, Burundi, Ethiopia, Madagascar, Nepal, Cambodia, and Timor-Leste were all expected to experience an increase of over 125% in the overweight prevalence among the poor. Fig 4B suggests that from 2016 to 2040, the largest increase in the share of the overweight that is relatively poor was in Ethiopia (36.1% [22.2%–50.8%]), Myanmar (27.6% [19.7%–34.6%]), and India (25.0% [18.2%–30.6%]). Finally, as shown in Fig 4C, the largest population growth of overweight and relatively poor individuals was projected to occur in Niger (from 189,000 to 1.05 million [1.04–1.07], 450.3%–466.1% growth) and Nigeria (from 3.05 to 10.92 million [10.77–11.09], 253.1%–263.6% growth). We projected that the number of individuals who are poor and overweight in India will grow from 14.32 to 34.50 million (34.03–35.12 million) and in Pakistan will grow from 5.23 to 13.29 million (13.22–13.39 million) over the same time period.

## Discussion

This work documented that, as per capita GDPs increased from $10,000 to $50,000, the prevalence of overweight and obesity shifts from concentrating predominantly among individuals in the highest to those in all deciles of personal wealth. In our adjusted specifications, we noted that the rich no longer had the highest overweight rates at a GDP per capita of $10,000, around the current GDP per capita of Namibia and Paraguay. We also identified another milestone of the wealth-overweight transition, around a GDP per capita of $50,000, where the wealthiest decile was less likely than the poorest decile to be overweight. A similar transition occurred in obesity across a GDP per capita range from $8,000 to $25,000. By 2040, 137 countries (125–149) were projected to reach $8,000 and enter the wealth-overweight transition zone.

As countries develop economically, we observed that, along with rising overweight rates, a larger fraction of the global overweight and obese populations become relatively poor. This is important for policy makers, as public health systems may be asked to shoulder the health and social consequences of overweight and obesity among those unable to cover the costs themselves. The relatively poor in many countries have limited access to healthcare and worse health outcomes, and this transition is one of both growing average burden and increasing health inequities. The intuitive implications are that cardiometabolic diseases and related conditions associated with overweight and obesity could be shifting to the relatively poor, and this has been occasionally documented [21,22].

Moreover, we found that the prevalence of overweight and obesity in the wealthiest decile had changed relatively little with increasing GDP per capita during the last 20 years. This arguably unexpected finding would be consistent with a pattern in which individuals in wealthier strata are less affected by the mechanisms influencing the rise in overweight and obesity among the poor as countries develop economically. One hypothesis for future work is that the wealthy may not change their food consumption as much as the poor in response to food prices, or their physical activity levels may not change much with labor choices as economic development occurs. It is also possible that the poor face changing supply-side factors (for example, food deserts or food swamps) as economic development occurs [23], while the rich do not.

Our projections, while speculative, suggest that if current trends and relationships continue, the rise in the number of relatively poor individuals who are also overweight will be most pronounced in low- and lower-middle-income countries over the next decade. Countries with currently very low GDP per capita, particularly those in sub-Saharan Africa, are not projected to realize a full wealth-overweight transition by 2040, yet there will still be substantial growth in overweight burden. We projected that lower-middle- and upper-middle-income countries will experience the shift in obesity burden as their GDP per capita increases above $8,000. In addition, lower-income countries are projected to experience population growth not expected in higher-income countries [20]. As life expectancy continues to rise, especially in lower-income countries, one implication of this study is that the growing burden of obesity among the poor will translate to increased reliance on public health systems for cardiovascular disease, type 2 diabetes, and related chronic conditions [9].

These projections highlight that policy makers, facing a growing burden of overweight-related diseases among the relatively poor, will need to design and implement interventions for a different target population than in the past. Specifically, as economic development and globalization progress in low-income countries, overweight-related care may be more widely demanded by the relatively poor. If governments want to tackle the burden of overweight and associated conditions, they must thus target their interventions at the poorest, which may require different approaches and financing than if they were targeting the wealthy. By planning for this reversal of the wealth gradient and subsequently different interventions, policy makers will use public health resources in a cost-effective manner, in addition to reducing health disparities.

Previous evidence suggests that sources of calorie intake differ across the relatively rich and poor, with the poorest having lower-quality diets than the richest [24]. While there are many possible explanations for this, if within a particular country, most of the health and economic burden of overweight and obesity is concentrated in the poor, then national policy makers would focus on the sources of calorie intake that the poor are most likely to consume. The same would be true in a different country where the burden is concentrated in the relatively rich population. There are multiple policy instruments that might be effective: tackling food deserts in low-income neighborhoods [23]; subsidizing fruit and vegetables [25]; taxing sugar (with a word of caution that this could be seen as a regressive tax with potentially positive health effects but negative effects on income inequality) [26]; working with industry to reformulate their products (where the industry and products on which a country would focus are distinct if overweight is concentrated in the rich or the poor) [27]; even potentially ensuring safe places for people in low-income neighborhoods to exercise and be outside [28].

From a public health perspective, anticipating the disease burden is of the utmost importance. This is true universally, and especially in resource scarce environments. This research and the accompanying wealth-overweight transition highlight when overweight and obesity burdens are expected to grow, and therefore equips domestic public health programs with key information needed regarding when to act and what to prioritize. This same information is useful to health donors concentrated on efficiently disbursing resources for the largest health gain possible, in the moment and when planning for the future.

This study has several limitations. First, although the World Health Surveys rely mostly on in-person surveys, which may reduce bias, its anthropometric measures are self-reported. Sensitivity analyses in Fig C and Tables H, J, and L in S1 Appendix suggest that this does not represent a significant source of bias, but ideally the outcome variable would be recorded anthropometrically in all instances. By including mostly self-reported data for high-income countries, we might observe lower BMI values in these countries, but comparisons of self-reported and national estimates suggest the bias averages to zero, and an indicator variable for self-reported data suggests it is only lower by 1.5% on average. Moreover, our results remain similar using only the Demographic and Health Surveys. Second, all surveys in this analysis are cross-sectional, so we cannot follow individuals over time to observe their changing personal wealth and anthropometric measurements. Third, there is a complex relationship between overweight, obesity, and personal wealth. Obesity may limit one's ability to attain wealth [29], or it may be itself an indication of wealth [30,31], depending on context. Fourth, expected overweight and obesity projections are based on a relatively simple model and highlight how expected changes in wealth may translate to overweight and obesity rates, given past trends and relationships continuing in the future. Some important determinants of overweight and obesity are not observed in our data. Reduced physical activity—as a result of changes in occupation, urbanization, transportation, or obesity status—may be responsible for weight gain among many populations. Similarly, we do not observe food prices and food availability at an individual level. Moreover, these variables are not projected to 2040 and thus cannot be used for our projections. For this reason, we use data and parameter uncertainty to construct a possible range of projections based on past trends and relationships with GDP per capita.

As overweight and obesity continue to rise globally, national policy makers may need to anticipate increased healthcare utilization by patients with overweight-related comorbidities. Little attention to date has been spent on understanding effective interventions in lower-middle- and upper-middle-income countries [32]. In high-income countries, public health measures to reduce overweight and obesity such as food labelling in stores and restaurants, media campaigns to change consumer behavior, and restrictions on unhealthy food advertising have gained traction [33]. By 2040, we predict 70.2% (64.1%–76.4%) of countries will begin or have undergone the wealth-overweight transition. By acting now, national public health programs can better plan interventions to control overweight and obesity, potentially reducing future disparities as well as avoiding future costs on strained public healthcare systems.

## Supporting information

S1 Checklist STROBE and RECORD checklists. We report how our study records information requested by the STROBE and RECORD checklists in this document.
(DOCX)

S1 Appendix Supplementary appendix. This supplementary appendix contains detailed information about our data and methods, in addition to multiple sensitivity analyses.
(PDF)

## Abbreviations

BMI: body mass index
GDP: gross domestic product
STROBE: Strengthening the Reporting of Observational Studies in Epidemiology
