# Supplementary material for: The overweight and obesity transition from the wealthy to the poor in low- and middle-income countries: A survey of household data from 103 countries
Source: PLoS Med. 2019 Nov 27;16(11):e1002968. doi: 10.1371/journal.pmed.1002968 (PMC6880978; doi:10.1371/journal.pmed.1002968)
Supplement: S1 Appendix — This supplementary appendix contains detailed information about our data and methods, in addition to multiple sensitivity analyses. (PDF) [file pmed.1002968.s002.pdf]

Appendix

1 Introduction

The purpose of this Appendix is to provide technical details on our data sources and methodology. Section 2 includes information on all data sources. Section 3 describes how we constructed personal wealth indices for our analysis. Section 4 discusses the use of self-reported data. Section 5 reports details on all fixed-effects regression results. Section 6 reports robustness checks to our estimation of the wealth-obesity and wealth-overweight transition zones. Section 7 describes an alternate meta-regression specification. Section 8 describes the forecasting methodology and out-of-sample validation.

2 Data

2.1 Data Identification

We searched the Global Health Data Exchange, a catalog of health-related data maintained by the Institute for Health Metrics and Evaluation, for individual-level data from surveys, censuses, and other data collection efforts containing personal wealth and body mass measures. Of 2,272 records identified via the Global Health Data Exchange, 1,018 records are not publicly available, with no application available, including reports and research papers. 463 records are cluster-level tabulations of individual data. 689 records require registration and applications (236 of which are the Demographic and Health Surveys and 233 are Multiple Indicator Cluster Surveys). 75 records are available for download (Living Standards Measurement Study, Reproductive Health Survey, United States National Health and Nutrition Examination Survey, etc). 634 records explicitly include individual height and weight measurements.

Data extraction efforts were focused on surveys that has cross-country comparable asset indicators and height and weight measurements. The Demographic and Health Surveys and World Health Surveys are cross-country surveys with consistent asset indicators, thus we focused on these surveys. Height and weight measurements were not administered for every Living Standards Measurement Study, and geographic variability was not improved by their inclusion. Eurobarometer and International Social Survey Programme surveys are self-reported height and weight data and do not improve the scope of the study significantly beyond the World Health Surveys. The Survey of Health, Aging, and Retirement in Europe includes individuals 50 and older which does not overlap with the Demographic and Health Surveys.

2.2 Demographic Health Surveys

The Demographic and Health Survey is a household-based, cross-sectional survey that is available for 90 countries, with over 200 country-years of data. Households are selected via multi-stage, stratified, probabilistic sampling design. Since 1984, the survey has collated data on individual and household attributes in low- and middle-income countries via standardized questionnaires. Most individual questionnaires restrict data collection to women, aged 15-49 years old, however there are also questionnaires for men and children. Since 1986, the survey has included anthropometric measurements, and since 1999, it has formally included a country wealth index. Although surveys in earlier years did not include as many questions as in subsequent years, the surveys provide cross-country comparable measurements of household attributes including, wealth asset indicators and anthropometric measurements.

2.3 World Health Surveys

The World Health Survey is a household-based, cross-sectional survey that is available for 70 countries, in the time period 2002-2004. Households are selected via a probabilistic sampling design. There are questionnaires specifically for low- and middle-income countries, and a similar but distinct questionnaire for high-income countries. Height and weight information are self-reported, but collected via a face-to-face interview. Wealth assets are standardized across low- and middle-income countries, with substantial overlap of wealth asset questions in high-income countries.

2.4 Institute for Health Metrics and Evaluation Gross Domestic Product (GDP) per capita

GDP per capita data were extracted from the Institute for Health Metrics and Evaluation. All data are in purchasing power parity 2017 dollars, and span 188 countries over 1950 to 2040. The Institute for Health Metrics and Evaluation uses four GDP per capita series to construct their estimates: (1) The International Monetary Fund's World Economic Outlook; (2) The World Bank's World Development Indicators; (3) The University of Pennsylvania Center for International Comparisons of Production, Income, and Prices GDP per capita series; and (4) Angus Madison's GDP per capita series hosted at the University of Groningen. The Institute for Health Metrics and Evaluation's methodology for combining these sources to produce one series is described in Spencer et al (2012) cited in the main text. Their methodology for projecting GDP per capita to 2040 is described in Dieleman et al (2018) also cited in the main text.

We use these data for the following reasons. First, it is estimated in purchasing power parity dollars, which are preferable over US dollars for estimating the effects of within-country wealth on obesity. Second, in addition to compiling four data sources to estimate past GDP per capita, the Institute for Health Metrics and Evaluation also incorporates uncertainty in the forecast, producing 1,000 "draws", or possible GDP per capita forecasts based on past data volatility and trends. We use these draws in our projections as well to capture uncertainty in future economic growth.

It is worth noting our choice of metrics for personal and national wealth. First, we intentionally chose a relative measure of personal wealth because we believe that is relevant for national policymakers, who are considering the health of the poor within their own countries. We estimate how obesity changes for a single wealth decile in a single country as that countryâĂŹs GDP changes. The focus is on the ordinal ordering rather than finding a measure of distance in personal wealth space. Second, we conduct additional analyses in the Supplementary Appendix Section 5 using an absolute measure of personal wealth, constructed with commonly found assets across the surveys. This measure would capture the distance between households that a Gini coefficient would also capture. The results from this analysis in the Supplementary Appendix, Tables S7 âĂŞ S12, reinforce the findings of the main text.

2.5 United Nations World Population Prospects

The UN Population World Population Prospect produces population estimates dis-aggregated by five-year age bins, sex, country, and year from 1950 until 2100. The World Population Prospect uses country-reported demographic data to complete its estimates from 1950-2015. To project population estimates to 2100, their models consider overall life-expectancy, migration flows, and fertility transitions. They consider the following fertility transition scenarios: medium-fertility assumption; high-fertility assumption; low-fertility assumption; constant-fertility assumption; instant-replacement assumption.

For this analysis, we extracted the medium-fertility variant. This means that future populations were projected with the following assumptions. In countries with high total fertility rates, fertility will continue to decline, stabilizing at two children per woman. Stated government policies and past trends of migration will continue into the future. Past trends in mortality rates will continue to into the future.

2.6 Construction of the Sample for Analysis

On the next page is a graphical representation of how potential survey respondents were excluded from the analysis. It displays how we arrived at a sample size of 2.24 million respondents.

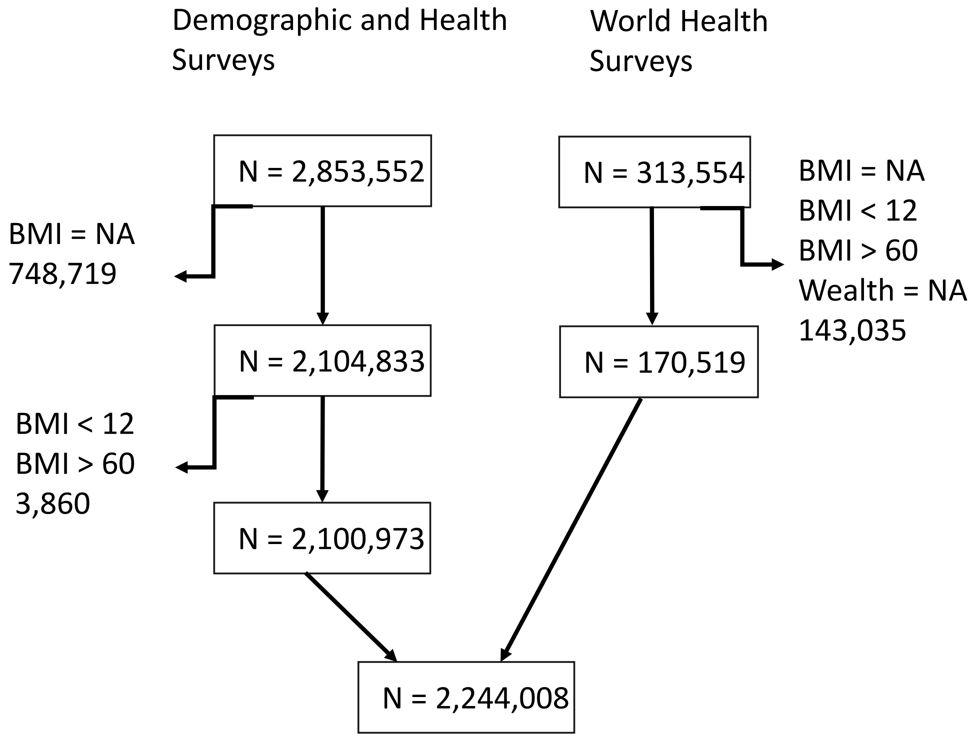
Demographic and Health Surveys
World Health Surveys
N = 2,853,552
N = 313,554
BMI = NA
748,719
BMI = NA
BMI < 12
BMI > 60
Wealth = NA
143,035
N = 2,104,833
N = 170,519
BMI < 12
BMI > 60
3,860
N = 2,100,973
N = 2,244,008

2.7 Descriptive Statistics

Descriptive statistics of all variables are summarized in Tables A, B, and C.

Table A: Full Sample Summary Statistics

Statistic	N	Mean	St. Dev.	Min	Pctl(25)	Pctl(75)	Max
Year	2, 244, 008	2010.29	4.667	1995	2005	2015	2016
Sex	2, 243, 991	1.958	0.199	1.000	2.000	2.000	2.000
BMI	2, 244, 008	23.032	4.779	12.010	19.710	25.440	59.950
Obese	2, 244, 008	0.085	0.278	0	0	0	1
Overweight	2, 244, 008	0.276	0.447	0	0	1	1
GDP per capita	2, 244, 008	5, 385.910	4, 743.620	460.565	2, 606.510	5, 616.000	96, 907.200

Table B: Full sample Age Group Frequencies

	Age Group	Frequency
1	(15,20]	0.191
2	(20,25]	0.183
3	(25,30]	0.172
4	(30,35]	0.142
5	(35,40]	0.131
6	(40,45]	0.108
7	(45,50]	0.073

Table C: Full Sample Educational Attainment Frequency

	Educational Attainment	Frequency
0	No Education	0.243
1	Primary Education	0.247
2	Secondary Education	0.406
3	Tertiary Education	0.104

In Tables D, E, and F, we provide survey specific information. Table D details the survey country and year, as well as the number of subjects, and the age frequency breakdown for the survey by 4 groups: $age < 20$; $20 \leq age < 30$; $30 \leq age < 40$; $age > 40$. Table E provides information on the unadjusted means of obesity by wealth quintile for each survey (where wealth = 1 is the poorest quintile and wealth = 5 is the richest quintile). Table F provides information on the unadjusted means of overweight by wealth quintile for each survey. Additionally, we graphically display survey coverage in Figures A and B. Figure A is a map which displays the number of surveys per country included in the analysis. Figure B is a heatmap which, for each country over time, displays if a survey is available, and if so what the mean, unadjusted overweight prevalence was.

Table D: Survey Respondent Demographics

Location	Year	N	% Women	$a < 20$	$20 \leq a < 30$	$30 \leq a < 40$	$a > 40$
Albania	2008	7,520	1	0.200	0.234	0.263	0.303
Armenia	2005	6,301	1	0.173	0.299	0.220	0.309
Armenia	2015	5,898	1	0.121	0.326	0.308	0.245
Austria	2003	879	0.377	0.036	0.135	0.256	0.572
Azerbaijan	2006	8,147	1	0.177	0.291	0.258	0.274
Bangladesh	2004	11,306	1	0.148	0.370	0.285	0.197
Bangladesh	2007	10,833	1	0.122	0.374	0.296	0.207
Bangladesh	2011	17,400	1	0.113	0.383	0.280	0.224
Bangladesh	2014	17,683	1	0.113	0.364	0.300	0.223
Belgium	2003	892	0.447	0.038	0.178	0.196	0.587
Benin	2006	16,717	1	0.167	0.381	0.281	0.171
Benin	2011	16,117	1	0.175	0.359	0.296	0.170
Bolivia	2003	17,277	1	0.218	0.332	0.262	0.188
Bolivia	2008	16,453	1	0.207	0.328	0.265	0.200
Bosnia and Herzegovina	2003	1,015	0.421	0.022	0.173	0.184	0.621
Brazil	2003	4,389	0.461	0.049	0.223	0.251	0.476
Burkina Faso	2003	13,913	0.942	0.203	0.346	0.253	0.198
Burkina Faso	2010	8,451	1	0.196	0.364	0.272	0.169
Burundi	2010	4,580	1	0.252	0.368	0.226	0.154
Burundi	2016	8,580	1	0.230	0.362	0.250	0.158
Cambodia	2005	8,350	1	0.211	0.303	0.256	0.229
Cambodia	2010	9,327	1	0.210	0.343	0.227	0.220
Cambodia	2014	11,480	1	0.169	0.345	0.264	0.222
Cameroon	2004	5,181	1	0.249	0.373	0.232	0.146
Cameroon	2011	7,884	1	0.235	0.379	0.231	0.156
Chad	2003	3,296	0.489	0.063	0.328	0.257	0.352
Chad	2004	3,582	1	0.107	0.487	0.303	0.102
Chad	2014	11,301	1	0.215	0.361	0.264	0.160
Colombia	2005	38,457	1	0.249	0.291	0.250	0.210
Colombia	2010	49,637	1	0.253	0.279	0.242	0.226
Comoros	2003	197	0.457	0.056	0.228	0.173	0.543
Comoros	2012	5,157	1	0.240	0.360	0.256	0.143
Congo	2003	1,547	0.485	0.063	0.360	0.266	0.311
Congo	2005	6,858	1	0.220	0.388	0.253	0.139
Congo	2011	5,644	1	0.204	0.349	0.274	0.173
Cote d'Ivoire	2003	2,646	0.589	0.073	0.339	0.288	0.300
Cote d'Ivoire	2011	4,814	1	0.206	0.383	0.253	0.158
Croatia	2003	969	0.407	0.025	0.083	0.152	0.741
Czech Republic	2003	879	0.448	0.023	0.171	0.195	0.612
Dem. Rep. of the Congo	2007	4,726	1	0.203	0.391	0.246	0.160
Dem. Rep. of the Congo	2013	9,362	1	0.212	0.380	0.251	0.158
Denmark	2003	939	0.480	0.020	0.098	0.184	0.698
Dominican Republic	2003	2,945	0.508	0.044	0.252	0.247	0.457
Dominican Republic	2013	10,874	1	0.206	0.323	0.253	0.217
Ecuador	2003	2,037	0.448	0.056	0.222	0.262	0.460
Egypt	2005	19,280	1	0.044	0.348	0.327	0.280
Egypt	2008	16,410	1	0.038	0.360	0.318	0.283
Egypt	2014	21,554	1	0.034	0.358	0.349	0.259
Estonia	2003	951	0.360	0.022	0.131	0.152	0.694
Ethiopia	2003	869	0.716	0.136	0.371	0.255	0.238

Table D: Survey Respondent Demographics

Location	Year	N	% Women	$a < 20$	$20 \leq a < 30$	$30 \leq a < 40$	$a > 40$
Ethiopia	2005	6,643	1	0.233	0.370	0.238	0.159
Ethiopia	2011	15,970	1	0.232	0.376	0.245	0.147
Ethiopia	2016	14,855	1	0.220	0.367	0.266	0.147
Finland	2003	992	0.450	0.021	0.100	0.123	0.756
France	2003	900	0.406	0.031	0.180	0.239	0.550
Gabon	2012	5,539	1	0.220	0.333	0.253	0.195
Georgia	2003	2,725	0.423	0.033	0.159	0.159	0.649
Germany	2003	1,091	0.411	0.029	0.120	0.160	0.690
Ghana	2003	8,613	0.792	0.138	0.309	0.261	0.292
Ghana	2008	4,814	1	0.207	0.345	0.261	0.187
Ghana	2014	4,750	1	0.187	0.332	0.277	0.204
Greece	2003	912	0.509	0.020	0.136	0.184	0.660
Guatemala	2003	2,747	0.438	0.053	0.290	0.234	0.423
Guatemala	2014	24,193	1	0.223	0.330	0.259	0.188
Guinea	2005	3,962	1	0.205	0.308	0.292	0.195
Guinea	2012	4,715	1	0.232	0.342	0.251	0.175
Guyana	2009	4,736	1	0.201	0.289	0.273	0.238
Haiti	2005	5,254	1	0.255	0.351	0.216	0.178
Haiti	2012	9,422	1	0.245	0.357	0.226	0.171
Honduras	2005	19,246	1	0.228	0.352	0.246	0.175
Honduras	2011	22,346	1	0.230	0.338	0.257	0.175
Hungary	2003	1,366	0.424	0.021	0.165	0.136	0.678
India	2003	6,401	0.531	0.062	0.255	0.258	0.425
India	2005	118,722	1	0.192	0.348	0.275	0.185
India	2015	687,156	1	0.178	0.340	0.268	0.214
Ireland	2003	772	0.448	0.063	0.177	0.189	0.570
Israel	2003	1,035	0.432	0.021	0.219	0.227	0.532
Italy	2003	851	0.429	0.032	0.155	0.176	0.637
Jordan	2007	5,196	1	0.021	0.308	0.391	0.280
Jordan	2009	4,730	1	0.025	0.307	0.393	0.275
Jordan	2012	7,187	1	0.022	0.291	0.385	0.302
Kazakhstan	2003	4,073	0.348	0.014	0.177	0.276	0.533
Kenya	2003	11,550	0.808	0.166	0.359	0.245	0.229
Kenya	2008	8,308	1	0.208	0.377	0.250	0.165
Kenya	2014	14,422	1	0.192	0.368	0.270	0.169
Kyrgyzstan	2012	8,098	1	0.194	0.342	0.242	0.222
Laos	2003	4,835	0.470	0.056	0.259	0.281	0.404
Latvia	2003	728	0.349	0.041	0.140	0.159	0.659
Lesotho	2004	3,414	1	0.250	0.342	0.222	0.186
Lesotho	2009	3,960	1	0.244	0.353	0.231	0.172
Lesotho	2014	3,388	1	0.236	0.353	0.246	0.164
Liberia	2007	6,914	1	0.189	0.359	0.273	0.180
Liberia	2013	4,595	1	0.201	0.350	0.262	0.187
Luxembourg	2003	684	0.494	0.048	0.146	0.227	0.579
Madagascar	2003	7,806	1	0.190	0.348	0.266	0.195
Madagascar	2008	8,375	1	0.230	0.330	0.261	0.178
Malawi	2003	4,741	0.410	0.084	0.394	0.210	0.312
Malawi	2004	11,127	1	0.205	0.424	0.226	0.146
Malawi	2010	7,601	1	0.223	0.382	0.247	0.149
Malawi	2015	8,048	1	0.214	0.377	0.263	0.146
Malaysia	2003	4,805	0.466	0.036	0.225	0.276	0.464

Table D: Survey Respondent Demographics

Location	Year	N	% Women	$a < 20$	$20 \leq a < 30$	$30 \leq a < 40$	$a > 40$
Maldives	2009	5,666	1	0.017	0.405	0.336	0.242
Mali	2003	467		0.086	0.206	0.257	0.452
Mali	2006	14,271	1	0.211	0.361	0.255	0.173
Mali	2012	5,251	1	0.176	0.384	0.279	0.160
Mauritania	2003	2,785		0.076	0.245	0.246	0.433
Mauritius	2003	2,326	0.613	0.041	0.210	0.260	0.489
Mexico	2003	23,450	0.422	0.053	0.243	0.243	0.461
Moldova	2005	7,245	1	0.191	0.284	0.237	0.289
Morocco	1995	7,245	1	0.191	0.284	0.237	0.289
Morocco	2003	18,619	0.961	0.184	0.327	0.253	0.236
Mozambique	2003	11,705	1	0.210	0.376	0.246	0.168
Mozambique	2011	13,599	1	0.223	0.350	0.267	0.160
Myanmar	2003	5,851	0.436	0.043	0.225	0.251	0.481
Myanmar	2015	12,657	1	0.143	0.292	0.302	0.263
Namibia	2003	3,632	0.401	0.061	0.319	0.268	0.352
Namibia	2006	9,533	1	0.224	0.352	0.255	0.168
Namibia	2013	5,155	1	0.168	0.298	0.225	0.309
Nepal	2003	2,784	0.652	0.078	0.324	0.269	0.330
Nepal	2006	10,738	1	0.226	0.354	0.239	0.182
Nepal	2011	6,144	1	0.221	0.350	0.248	0.181
Nepal	2016	6,457	1	0.205	0.343	0.264	0.188
Netherlands	2003	1,041	0.320	0.137	0.169	0.109	0.585
Niger	2006	4,542	1	0.197	0.367	0.268	0.168
Niger	2012	5,109	1	0.169	0.375	0.300	0.155
Nigeria	2003	7,444	1	0.227	0.371	0.230	0.172
Nigeria	2008	32,358	1	0.195	0.372	0.254	0.178
Nigeria	2013	38,334	1	0.202	0.354	0.259	0.185
Norway	2003	946	0.495	0.031	0.153	0.214	0.603
Pakistan	2003	3,110	0.632	0.081	0.264	0.264	0.391
Pakistan	2012	4,676	1	0.045	0.351	0.344	0.260
Paraguay	2003	4,641	0.487	0.062	0.262	0.230	0.446
Peru	2004	27,197	1	0.192	0.304	0.277	0.227
Peru	2007	27,197	1	0.192	0.304	0.277	0.227
Peru	2009	23,336	1	0.190	0.305	0.279	0.226
Peru	2010	22,530	1	0.191	0.296	0.285	0.228
Peru	2011	22,246	1	0.187	0.295	0.283	0.235
Peru	2012	23,672	1	0.188	0.293	0.282	0.237
Philippines	2003	8,143	0.486	0.045	0.260	0.288	0.407
Portugal	2003	862	0.414	0.037	0.167	0.188	0.608
Russia	2003	3,437	0.356	0.024	0.138	0.165	0.672
Rwanda	2005	5,642	1	0.233	0.349	0.240	0.178
Rwanda	2010	6,952	1	0.223	0.383	0.231	0.163
Rwanda	2014	6,698	1	0.208	0.360	0.272	0.161
Sao Tome and Principe	2008	2,386	1	0.205	0.342	0.259	0.195
Senegal	2003	790		0.075	0.299	0.234	0.392
Senegal	2005	4,580	1	0.257	0.356	0.231	0.156
Senegal	2010	5,745	1	0.237	0.366	0.243	0.154
Sierra Leone	2008	3,501	1	0.166	0.379	0.293	0.162
Sierra Leone	2013	7,983	1	0.230	0.325	0.281	0.163
Slovakia	2003	1,667		0.047	0.347	0.179	0.427
Slovenia	2003	561	0.469	0.036	0.185	0.164	0.615

Table D: Survey Respondent Demographics

Location	Year	N	% Women	$a < 20$	$20 \leq a < 30$	$30 \leq a < 40$	$a > 40$
South Africa	2003	350	0.443	0.051	0.266	0.309	0.374
Spain	2003	5,979	0.417	0.017	0.104	0.172	0.707
Sri Lanka	2003	4,040	0.502	0.048	0.219	0.244	0.488
Swaziland	2003	1,756	0.428	0.085	0.305	0.246	0.364
Swaziland	2006	4,856	1	0.256	0.352	0.227	0.165
Sweden	2003	936	0.422	0.028	0.128	0.162	0.682
Tajikistan	2012	9,627	1	0.207	0.359	0.234	0.200
Tanzania	2004	10,232	1	0.222	0.367	0.251	0.160
Tanzania	2010	10,038	1	0.219	0.342	0.261	0.178
Tanzania	2015	13,158	1	0.221	0.346	0.254	0.179
Timor-Leste	2009	12,851	1	0.244	0.320	0.246	0.190
Timor-Leste	2016	12,443	1	0.247	0.315	0.235	0.203
Togo	2013	4,827	1	0.188	0.343	0.280	0.189
Tunisia	2003	4,111	0.506	0.052	0.236	0.256	0.456
Turkey	2003	3,288	1	0.035	0.565	0.347	0.053
Uganda	2006	2,860	1	0.223	0.367	0.261	0.149
Uganda	2011	2,704	1	0.234	0.375	0.250	0.141
Uganda	2016	6,049	1	0.228	0.369	0.247	0.155
Ukraine	2003	1,293	0.339	0.026	0.162	0.184	0.628
United Arab Emirates	2003	1,022	0.525	0.044	0.248	0.321	0.387
United Kingdom	2003	995	0.371	0.030	0.124	0.222	0.624
Uruguay	2003	2,913	0.487	0.024	0.188	0.203	0.585
Vietnam	2003	3,143	0.448	0.050	0.192	0.286	0.472
Zambia	2003	2,162	0.464	0.075	0.351	0.269	0.305
Zambia	2007	7,046	1	0.223	0.390	0.248	0.140
Zambia	2013	16,231	1	0.225	0.355	0.269	0.151
Zimbabwe	2003	2,285	0.414	0.091	0.353	0.240	0.316
Zimbabwe	2005	8,717	1	0.240	0.379	0.231	0.150
Zimbabwe	2010	8,789	1	0.215	0.384	0.253	0.148
Zimbabwe	2015	9,650	1	0.218	0.346	0.281	0.156

Table E: Unadjusted Obesity by Wealth Quintile

Location	Year	Wealth=1	Wealth=2	Wealth=3	Wealth=4	Wealth=5
Albania	2008	0.070	0.107	0.118	0.102	0.091
Armenia	2005	0.123	0.175	0.176	0.179	0.125
Armenia	2015	0.162	0.167	0.163	0.143	0.127
Austria	2003	0.097	0.101	0.109	0.153	0.109
Azerbaijan	2006	0.113	0.148	0.172	0.213	0.211
Bangladesh	2004	0.003	0.003	0.005	0.014	0.057
Bangladesh	2007	0.002	0.001	0.011	0.018	0.071
Bangladesh	2011	0.004	0.008	0.014	0.032	0.098
Bangladesh	2014	0.008	0.015	0.027	0.049	0.119
Belgium	2003	0.101	0.128	0.123	0.140	0.096
Benin	2006	0.012	0.017	0.030	0.065	0.144
Benin	2011	0.022	0.038	0.053	0.084	0.133
Bolivia	2003	0.072	0.142	0.170	0.202	0.171
Bolivia	2008	0.100	0.163	0.201	0.239	0.163
Bosnia and Herzegovina	2003	0.138	0.118	0.099	0.069	0.109
Brazil	2003	0.118	0.108	0.097	0.108	0.096
Burkina Faso	2003	0.004	0.001	0.003	0.015	0.112
Burkina Faso	2010	0.005	0.006	0.006	0.029	0.104
Burundi	2010	0.002	0.003	0.009	0.012	0.082
Burundi	2016	0.001	0.003	0.006	0.010	0.089
Cambodia	2005	0.001	0.006	0.005	0.009	0.031
Cambodia	2010	0.003	0.007	0.010	0.025	0.023
Cambodia	2014	0.013	0.025	0.025	0.038	0.040
Cameroon	2004	0.015	0.032	0.079	0.115	0.154
Cameroon	2011	0.019	0.050	0.097	0.161	0.186
Chad	2003	0.129	0.094	0.136	0.132	0.089
Chad	2004	0.007	0.005	0.010	0.028	0.067
Chad	2014	0.011	0.008	0.011	0.013	0.070
Colombia	2005	0.096	0.119	0.117	0.124	0.117
Colombia	2010	0.133	0.163	0.157	0.140	0.127
Comoros	2003	0.024	0.053	0.025	0.026	0.026
Comoros	2012	0.094	0.111	0.134	0.128	0.142
Congo	2003	0.068	0.045	0.039	0.026	0.045
Congo	2005	0.020	0.038	0.067	0.085	0.162
Congo	2011	0.020	0.026	0.056	0.083	0.130
Cote d'Ivoire	2003	0.058	0.040	0.040	0.040	0.032
Cote d'Ivoire	2011	0.009	0.031	0.068	0.100	0.104
Croatia	2003	0.155	0.119	0.179	0.222	0.156
Czech Republic	2003	0.215	0.206	0.199	0.193	0.171
Democratic Republic of the Congo	2007	0.010	0.012	0.010	0.024	0.064
Democratic Republic of the Congo	2013	0.005	0.005	0.010	0.026	0.097
Denmark	2003	0.149	0.138	0.106	0.160	0.102
Dominican Republic	2003	0.166	0.171	0.160	0.126	0.127
Dominican Republic	2013	0.161	0.204	0.216	0.222	0.205
Ecuador	2003	0.135	0.118	0.074	0.108	0.108
Egypt	2005	0.302	0.370	0.443	0.521	0.562
Egypt	2008	0.278	0.344	0.402	0.420	0.428
Egypt	2014	0.430	0.463	0.464	0.502	0.460
Estonia	2003	0.178	0.215	0.221	0.136	0.166
Ethiopia	2003	0.034	0.011	0.017	0.006	0.029
Ethiopia	2005	0.008	0.004	0.002	0.005	0.037

Table E: Unadjusted Obesity by Wealth Quintile

Location	Year	Wealth=1	Wealth=2	Wealth=3	Wealth=4	Wealth=5
Ethiopia	2011	0.004	0.004	0.002	0.011	0.047
Ethiopia	2016	0.003	0.003	0.002	0.016	0.086
Finland	2003	0.146	0.167	0.186	0.192	0.121
France	2003	0.061	0.100	0.086	0.057	0.061
Gabon	2012	0.064	0.115	0.172	0.209	0.223
Georgia	2003	0.106	0.095	0.118	0.103	0.114
Germany	2003	0.105	0.170	0.174	0.142	0.138
Ghana	2003	0.005	0.021	0.065	0.148	0.188
Ghana	2008	0.018	0.031	0.051	0.117	0.203
Ghana	2014	0.016	0.048	0.109	0.190	0.294
Greece	2003	0.142	0.172	0.184	0.185	0.177
Guatemala	2003	0.120	0.110	0.118	0.120	0.064
Guatemala	2014	0.100	0.150	0.220	0.244	0.272
Guinea	2005	0.010	0.008	0.015	0.031	0.074
Guinea	2012	0.008	0.010	0.040	0.060	0.106
Guyana	2009	0.162	0.228	0.204	0.242	0.226
Haiti	2005	0.011	0.020	0.039	0.073	0.133
Haiti	2012	0.013	0.037	0.051	0.086	0.155
Honduras	2005	0.072	0.110	0.193	0.236	0.237
Honduras	2011	0.096	0.155	0.234	0.265	0.272
Hungary	2003	0.212	0.194	0.223	0.198	0.198
India	2003	0.014	0.018	0.021	0.020	0.027
India	2005	0.003	0.008	0.019	0.051	0.095
India	2015	0.008	0.018	0.036	0.069	0.107
Ireland	2003	0.160	0.201	0.175	0.155	0.098
Israel	2003	0.043	0.106	0.106	0.130	0.198
Italy	2003	0.099	0.094	0.053	0.106	0.059
Jordan	2007	0.291	0.276	0.293	0.319	0.302
Jordan	2009	0.432	0.334	0.400	0.419	0.336
Jordan	2012	0.392	0.405	0.347	0.374	0.365
Kazakhstan	2003	0.091	0.130	0.123	0.113	0.150
Kenya	2003	0.005	0.018	0.048	0.094	0.154
Kenya	2008	0.014	0.032	0.067	0.110	0.136
Kenya	2014	0.015	0.046	0.063	0.114	0.193
Kyrgyzstan	2012	0.140	0.128	0.113	0.112	0.097
Laos	2003	0.008	0.010	0.008	0.010	0.028
Latvia	2003	0.199	0.240	0.213	0.185	0.200
Lesotho	2004	0.075	0.111	0.149	0.197	0.250
Lesotho	2009	0.071	0.133	0.139	0.168	0.297
Lesotho	2014	0.078	0.160	0.182	0.267	0.262
Liberia	2007	0.019	0.023	0.036	0.079	0.103
Liberia	2013	0.030	0.047	0.050	0.064	0.130
Luxembourg	2003	0.153	0.182	0.124	0.109	0.125
Madagascar	2003	0.004	0.004	0.010	0.024	0.023
Madagascar	2008	0.003	0.001	0.003	0.013	0.041
Malawi	2003	0.084	0.093	0.077	0.056	0.036
Malawi	2004	0.006	0.006	0.018	0.021	0.061
Malawi	2010	0.013	0.027	0.012	0.023	0.098
Malawi	2015	0.016	0.026	0.035	0.065	0.140
Malaysia	2003	0.102	0.117	0.118	0.117	0.107
Maldives	2009	0.120	0.125	0.117	0.129	0.143

Table E: Unadjusted Obesity by Wealth Quintile

Location	Year	Wealth=1	Wealth=2	Wealth=3	Wealth=4	Wealth=5
Mali	2003	0.289	0.227	0.200	0.140	0.168
Mali	2006	0.009	0.019	0.028	0.070	0.123
Mali	2012	0.012	0.022	0.020	0.073	0.144
Mauritania	2003	0.109	0.110	0.102	0.114	0.129
Mauritius	2003	0.077	0.080	0.084	0.078	0.086
Mexico	2003	0.130	0.154	0.168	0.177	0.170
Moldova	2005	0.178	0.206	0.189	0.180	0.135
Morocco	1995	0.178	0.206	0.189	0.180	0.135
Morocco	2003	0.043	0.074	0.095	0.149	0.161
Mozambique	2003	0.005	0.007	0.009	0.047	0.141
Mozambique	2011	0.003	0.007	0.020	0.059	0.169
Myanmar	2003	0.029	0.021	0.011	0.010	0.006
Myanmar	2015	0.028	0.039	0.053	0.062	0.093
Namibia	2003	0.088	0.084	0.116	0.128	0.154
Namibia	2006	0.027	0.060	0.113	0.161	0.196
Namibia	2013	0.021	0.077	0.114	0.192	0.227
Nepal	2003	0.023	0.013	0.012	0.031	0.059
Nepal	2006	0	0.002	0.001	0.010	0.030
Nepal	2011	0.007	0.005	0.013	0.023	0.067
Nepal	2016	0.006	0.021	0.019	0.047	0.130
Netherlands	2003	0.129	0.115	0.139	0.130	0.135
Niger	2006	0.008	0.005	0.018	0.039	0.133
Niger	2012	0.012	0.019	0.014	0.038	0.151
Nigeria	2003	0.018	0.027	0.049	0.059	0.129
Nigeria	2008	0.018	0.022	0.041	0.069	0.127
Nigeria	2013	0.021	0.030	0.059	0.095	0.167
Norway	2003	0.074	0.105	0.101	0.063	0.079
Pakistan	2003	0.077	0.087	0.103	0.121	0.132
Pakistan	2012	0.040	0.081	0.135	0.213	0.255
Paraguay	2003	0.070	0.099	0.117	0.105	0.165
Peru	2004	0.053	0.129	0.168	0.177	0.154
Peru	2007	0.053	0.129	0.168	0.177	0.154
Peru	2009	0.077	0.131	0.183	0.198	0.172
Peru	2010	0.074	0.126	0.179	0.193	0.183
Peru	2011	0.073	0.152	0.207	0.215	0.169
Peru	2012	0.087	0.156	0.212	0.223	0.191
Philippines	2003	0.029	0.037	0.050	0.047	0.061
Portugal	2003	0.098	0.174	0.191	0.211	0.176
Russia	2003	0.129	0.149	0.170	0.174	0.149
Rwanda	2005	0.006	0.004	0.007	0.007	0.034
Rwanda	2010	0.006	0.006	0.012	0.022	0.069
Rwanda	2014	0.004	0.023	0.015	0.038	0.132
Sao Tome and Principe	2008	0.096	0.099	0.084	0.148	0.144
Senegal	2003	0.044	0.057	0.070	0.063	0.045
Senegal	2005	0.018	0.024	0.058	0.106	0.109
Senegal	2010	0.027	0.027	0.042	0.060	0.092
Sierra Leone	2008	0.097	0.053	0.060	0.118	0.140
Sierra Leone	2013	0.022	0.018	0.030	0.057	0.115
Slovakia	2003	0.155	0.118	0.112	0.100	0.114
Slovenia	2003	0.168	0.143	0.134	0.125	0.134

Table E: Unadjusted Obesity by Wealth Quintile

Location	Year	Wealth=1	Wealth=2	Wealth=3	Wealth=4	Wealth=5
South Africa	2003	0.211	0.191	0.310	0.254	0.435
Spain	2003	0.180	0.167	0.185	0.149	0.131
Sri Lanka	2003	0.032	0.044	0.031	0.038	0.051
Swaziland	2003	0.353	0.294	0.249	0.305	0.248
Swaziland	2006	0.140	0.191	0.226	0.265	0.339
Sweden	2003	0.085	0.111	0.125	0.102	0.102
Tajikistan	2012	0.057	0.086	0.096	0.109	0.131
Tanzania	2004	0.008	0.010	0.018	0.043	0.124
Tanzania	2010	0.008	0.016	0.037	0.073	0.167
Tanzania	2015	0.018	0.034	0.061	0.144	0.229
Timor-Leste	2009	0.003	0.005	0.004	0.007	0.017
Timor-Leste	2016	0.007	0.007	0.010	0.021	0.033
Togo	2013	0.024	0.036	0.065	0.145	0.219
Tunisia	2003	0.053	0.085	0.073	0.106	0.103
Turkey	2003	0.201	0.237	0.255	0.242	0.194
Uganda	2006	0	0.006	0.014	0.037	0.118
Uganda	2011	0.014	0.015	0.028	0.058	0.110
Uganda	2016	0.006	0.024	0.039	0.078	0.175
Ukraine	2003	0.158	0.202	0.197	0.143	0.178
United Arab Emirates	2003	0.200	0.221	0.146	0.207	0.162
United Kingdom	2003	0.156	0.181	0.186	0.146	0.236
Uruguay	2003	0.122	0.125	0.143	0.171	0.139
Vietnam	2003	0.003	0.002	0	0	0.003
Zambia	2003	0.111	0.063	0.062	0.030	0.066
Zambia	2007	0.007	0.008	0.036	0.067	0.134
Zambia	2013	0.010	0.021	0.040	0.104	0.141
Zimbabwe	2003	0.138	0.115	0.103	0.077	0.072
Zimbabwe	2005	0.030	0.030	0.052	0.091	0.140
Zimbabwe	2010	0.040	0.075	0.084	0.138	0.178
Zimbabwe	2015	0.039	0.075	0.126	0.183	0.239

Table F: Unadjusted Overweight by Wealth Quintile

Location	Year	Wealth=1	Wealth=2	Wealth=3	Wealth=4	Wealth=5
Albania	2008	0.331	0.433	0.430	0.399	0.375
Armenia	2005	0.427	0.450	0.431	0.421	0.393
Armenia	2015	0.447	0.459	0.438	0.454	0.447
Austria	2003	0.455	0.449	0.414	0.455	0.451
Azerbaijan	2006	0.379	0.406	0.456	0.551	0.534
Bangladesh	2004	0.021	0.030	0.059	0.105	0.290
Bangladesh	2007	0.033	0.047	0.088	0.146	0.364
Bangladesh	2011	0.049	0.078	0.128	0.215	0.384
Bangladesh	2014	0.089	0.142	0.197	0.286	0.474
Belgium	2003	0.433	0.458	0.475	0.444	0.388
Benin	2006	0.079	0.105	0.140	0.236	0.370
Benin	2011	0.149	0.205	0.249	0.321	0.391
Bolivia	2003	0.360	0.487	0.506	0.506	0.470
Bolivia	2008	0.431	0.516	0.555	0.546	0.474
Bosnia and Herzegovina	2003	0.409	0.438	0.458	0.402	0.455
Brazil	2003	0.417	0.453	0.394	0.359	0.338
Burkina Faso	2003	0.023	0.028	0.043	0.094	0.328
Burkina Faso	2010	0.040	0.054	0.049	0.134	0.288
Burundi	2010	0.038	0.050	0.061	0.086	0.269
Burundi	2016	0.027	0.028	0.056	0.097	0.267
Cambodia	2005	0.041	0.051	0.066	0.127	0.183
Cambodia	2010	0.059	0.082	0.102	0.168	0.158
Cambodia	2014	0.120	0.164	0.179	0.232	0.232
Cameroon	2004	0.135	0.204	0.308	0.365	0.457
Cameroon	2011	0.121	0.229	0.362	0.421	0.483
Chad	2003	0.359	0.324	0.365	0.422	0.351
Chad	2004	0.043	0.049	0.064	0.118	0.260
Chad	2014	0.086	0.085	0.083	0.087	0.246
Colombia	2005	0.348	0.389	0.404	0.412	0.386
Colombia	2010	0.404	0.450	0.440	0.440	0.442
Comoros	2003	0.244	0.289	0.150	0.179	0.179
Comoros	2012	0.283	0.369	0.371	0.416	0.446
Congo	2003	0.265	0.239	0.165	0.197	0.205
Congo	2005	0.131	0.210	0.260	0.309	0.404
Congo	2011	0.112	0.129	0.174	0.227	0.395
Cote d'Ivoire	2003	0.254	0.211	0.244	0.298	0.230
Cote d'Ivoire	2011	0.124	0.169	0.236	0.348	0.370
Croatia	2003	0.562	0.546	0.533	0.572	0.510
Czech Republic	2003	0.610	0.611	0.580	0.528	0.497
Democratic Republic of the Congo	2007	0.058	0.067	0.061	0.154	0.248
Democratic Republic of the Congo	2013	0.075	0.086	0.101	0.171	0.321
Denmark	2003	0.468	0.527	0.471	0.492	0.380
Dominican Republic	2003	0.513	0.487	0.467	0.435	0.409
Dominican Republic	2013	0.418	0.483	0.506	0.532	0.528
Ecuador	2003	0.429	0.452	0.395	0.423	0.376
Egypt	2005	0.643	0.739	0.793	0.849	0.890
Egypt	2008	0.637	0.722	0.795	0.834	0.831
Egypt	2014	0.797	0.821	0.833	0.881	0.876
Estonia	2003	0.524	0.492	0.505	0.495	0.486
Ethiopia	2003	0.178	0.098	0.137	0.063	0.093

Table F: Unadjusted Overweight by Wealth Quintile

Location	Year	Wealth=1	Wealth=2	Wealth=3	Wealth=4	Wealth=5
Ethiopia	2005	0.020	0.030	0.019	0.055	0.184
Ethiopia	2011	0.024	0.024	0.024	0.072	0.237
Ethiopia	2016	0.033	0.037	0.034	0.099	0.296
Finland	2003	0.497	0.606	0.573	0.561	0.515
France	2003	0.300	0.311	0.323	0.310	0.222
Gabon	2012	0.244	0.341	0.427	0.478	0.505
Georgia	2003	0.451	0.433	0.476	0.456	0.500
Germany	2003	0.457	0.555	0.587	0.518	0.459
Ghana	2003	0.052	0.130	0.238	0.404	0.502
Ghana	2008	0.116	0.159	0.247	0.416	0.485
Ghana	2014	0.116	0.229	0.348	0.509	0.612
Greece	2003	0.552	0.554	0.547	0.598	0.629
Guatemala	2003	0.434	0.418	0.393	0.373	0.321
Guatemala	2014	0.392	0.457	0.532	0.594	0.606
Guinea	2005	0.074	0.076	0.094	0.157	0.293
Guinea	2012	0.058	0.118	0.171	0.277	0.344
Guyana	2009	0.461	0.460	0.433	0.513	0.518
Haiti	2005	0.091	0.130	0.172	0.246	0.356
Haiti	2012	0.116	0.162	0.247	0.282	0.391
Honduras	2005	0.289	0.387	0.477	0.541	0.540
Honduras	2011	0.368	0.462	0.540	0.555	0.571
Hungary	2003	0.562	0.527	0.634	0.542	0.538
India	2003	0.075	0.096	0.115	0.123	0.190
India	2005	0.024	0.060	0.121	0.214	0.342
India	2015	0.059	0.113	0.180	0.273	0.356
Ireland	2003	0.519	0.455	0.519	0.471	0.444
Israel	2003	0.382	0.391	0.435	0.430	0.440
Italy	2003	0.368	0.424	0.380	0.418	0.385
Jordan	2007	0.595	0.677	0.665	0.676	0.673
Jordan	2009	0.715	0.686	0.724	0.717	0.736
Jordan	2012	0.691	0.728	0.698	0.706	0.709
Kazakhstan	2003	0.365	0.442	0.482	0.436	0.474
Kenya	2003	0.086	0.108	0.205	0.347	0.444
Kenya	2008	0.093	0.145	0.246	0.356	0.423
Kenya	2014	0.113	0.202	0.267	0.395	0.504
Kyrgyzstan	2012	0.395	0.397	0.353	0.368	0.309
Laos	2003	0.062	0.077	0.077	0.121	0.188
Latvia	2003	0.555	0.627	0.518	0.493	0.483
Lesotho	2004	0.295	0.357	0.405	0.478	0.576
Lesotho	2009	0.260	0.369	0.394	0.444	0.565
Lesotho	2014	0.270	0.402	0.475	0.503	0.558
Liberia	2007	0.104	0.119	0.178	0.264	0.323
Liberia	2013	0.177	0.200	0.227	0.247	0.345
Luxembourg	2003	0.489	0.467	0.445	0.460	0.390
Madagascar	2003	0.035	0.049	0.097	0.129	0.156
Madagascar	2008	0.024	0.024	0.031	0.076	0.178
Malawi	2003	0.376	0.326	0.297	0.263	0.177
Malawi	2004	0.100	0.086	0.120	0.150	0.250
Malawi	2010	0.101	0.145	0.139	0.174	0.293
Malawi	2015	0.122	0.151	0.199	0.223	0.406
Malaysia	2003	0.305	0.360	0.368	0.396	0.392

Table F: Unadjusted Overweight by Wealth Quintile

Location	Year	Wealth=1	Wealth=2	Wealth=3	Wealth=4	Wealth=5
Maldives	2009	0.413	0.428	0.425	0.461	0.496
Mali	2003	0.578	0.477	0.463	0.376	0.396
Mali	2006	0.088	0.095	0.138	0.232	0.323
Mali	2012	0.111	0.107	0.116	0.234	0.355
Mauritania	2003	0.360	0.381	0.354	0.361	0.393
Mauritius	2003	0.324	0.344	0.341	0.334	0.340
Mexico	2003	0.465	0.516	0.542	0.551	0.561
Moldova	2005	0.425	0.430	0.424	0.414	0.370
Morocco	1995	0.425	0.430	0.424	0.414	0.370
Morocco	2003	0.241	0.299	0.369	0.431	0.456
Mozambique	2003	0.061	0.073	0.099	0.213	0.338
Mozambique	2011	0.063	0.092	0.143	0.264	0.417
Myanmar	2003	0.171	0.134	0.094	0.101	0.084
Myanmar	2015	0.150	0.206	0.240	0.288	0.346
Namibia	2003	0.269	0.260	0.317	0.325	0.385
Namibia	2006	0.099	0.197	0.288	0.363	0.437
Namibia	2013	0.137	0.238	0.314	0.444	0.445
Nepal	2003	0.106	0.101	0.091	0.165	0.257
Nepal	2006	0.028	0.035	0.049	0.082	0.236
Nepal	2011	0.032	0.055	0.093	0.181	0.309
Nepal	2016	0.093	0.155	0.131	0.225	0.420
Netherlands	2003	0.340	0.433	0.490	0.481	0.418
Niger	2006	0.051	0.048	0.100	0.189	0.406
Niger	2012	0.102	0.115	0.111	0.226	0.433
Nigeria	2003	0.111	0.125	0.193	0.234	0.381
Nigeria	2008	0.100	0.133	0.191	0.254	0.387
Nigeria	2013	0.112	0.160	0.231	0.319	0.430
Norway	2003	0.368	0.479	0.473	0.365	0.386
Pakistan	2003	0.256	0.286	0.300	0.349	0.411
Pakistan	2012	0.174	0.273	0.388	0.531	0.624
Paraguay	2003	0.295	0.374	0.412	0.408	0.492
Peru	2004	0.356	0.475	0.536	0.542	0.494
Peru	2007	0.356	0.475	0.536	0.542	0.494
Peru	2009	0.396	0.496	0.549	0.551	0.525
Peru	2010	0.396	0.495	0.538	0.562	0.522
Peru	2011	0.412	0.512	0.574	0.583	0.520
Peru	2012	0.437	0.545	0.587	0.593	0.554
Philippines	2003	0.163	0.198	0.215	0.204	0.267
Portugal	2003	0.422	0.581	0.566	0.589	0.509
Russia	2003	0.516	0.525	0.539	0.548	0.505
Rwanda	2005	0.095	0.085	0.110	0.112	0.241
Rwanda	2010	0.121	0.116	0.152	0.190	0.311
Rwanda	2014	0.123	0.166	0.179	0.261	0.419
Sao Tome and Principe	2008	0.268	0.309	0.341	0.402	0.395
Senegal	2003	0.239	0.220	0.247	0.266	0.250
Senegal	2005	0.108	0.112	0.203	0.290	0.296
Senegal	2010	0.116	0.134	0.164	0.228	0.305
Sierra Leone	2008	0.268	0.213	0.278	0.374	0.395
Sierra Leone	2013	0.110	0.133	0.168	0.226	0.309
Slovakia	2003	0.469	0.379	0.386	0.441	0.377
Slovenia	2003	0.540	0.473	0.491	0.482	0.464

Table F: Unadjusted Overweight by Wealth Quintile

Location	Year	Wealth=1	Wealth=2	Wealth=3	Wealth=4	Wealth=5
South Africa	2003	0.479	0.471	0.493	0.493	0.638
Spain	2003	0.577	0.566	0.601	0.530	0.513
Sri Lanka	2003	0.133	0.164	0.160	0.162	0.204
Swaziland	2003	0.681	0.686	0.599	0.610	0.623
Swaziland	2006	0.403	0.460	0.524	0.556	0.617
Sweden	2003	0.434	0.481	0.495	0.406	0.396
Tajikistan	2012	0.230	0.283	0.312	0.323	0.391
Tanzania	2004	0.093	0.106	0.124	0.209	0.374
Tanzania	2010	0.092	0.126	0.162	0.289	0.420
Tanzania	2015	0.129	0.184	0.250	0.383	0.492
Timor-Leste	2009	0.024	0.027	0.034	0.061	0.103
Timor-Leste	2016	0.053	0.063	0.087	0.144	0.160
Togo	2013	0.105	0.190	0.262	0.388	0.496
Tunisia	2003	0.303	0.330	0.380	0.427	0.438
Turkey	2003	0.533	0.574	0.623	0.616	0.546
Uganda	2006	0.059	0.078	0.149	0.202	0.334
Uganda	2011	0.056	0.097	0.181	0.269	0.380
Uganda	2016	0.077	0.145	0.215	0.277	0.434
Ukraine	2003	0.514	0.550	0.560	0.547	0.568
United Arab Emirates	2003	0.556	0.608	0.549	0.665	0.613
United Kingdom	2003	0.503	0.467	0.497	0.487	0.528
Uruguay	2003	0.433	0.503	0.487	0.514	0.472
Vietnam	2003	0.029	0.024	0.021	0.025	0.051
Zambia	2003	0.336	0.255	0.235	0.158	0.206
Zambia	2007	0.099	0.101	0.165	0.258	0.368
Zambia	2013	0.099	0.137	0.188	0.320	0.386
Zimbabwe	2003	0.416	0.405	0.338	0.326	0.283
Zimbabwe	2005	0.143	0.163	0.217	0.314	0.403
Zimbabwe	2010	0.188	0.260	0.297	0.394	0.441
Zimbabwe	2015	0.217	0.279	0.381	0.460	0.511

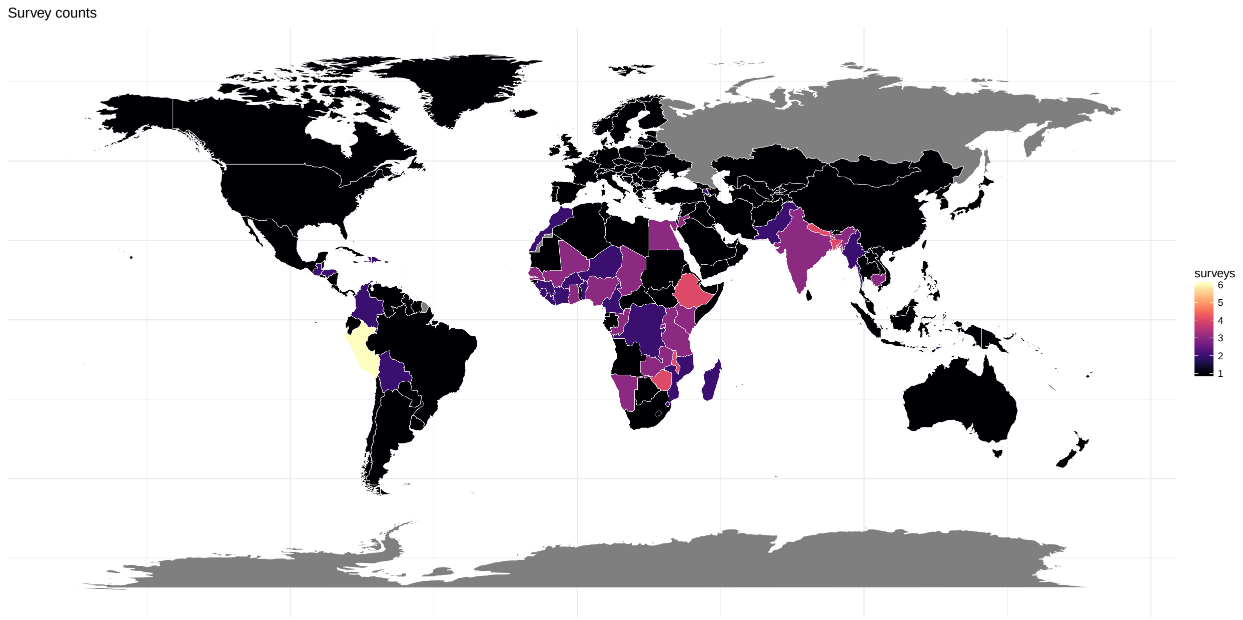

Figure A: Number of Surveys per Country

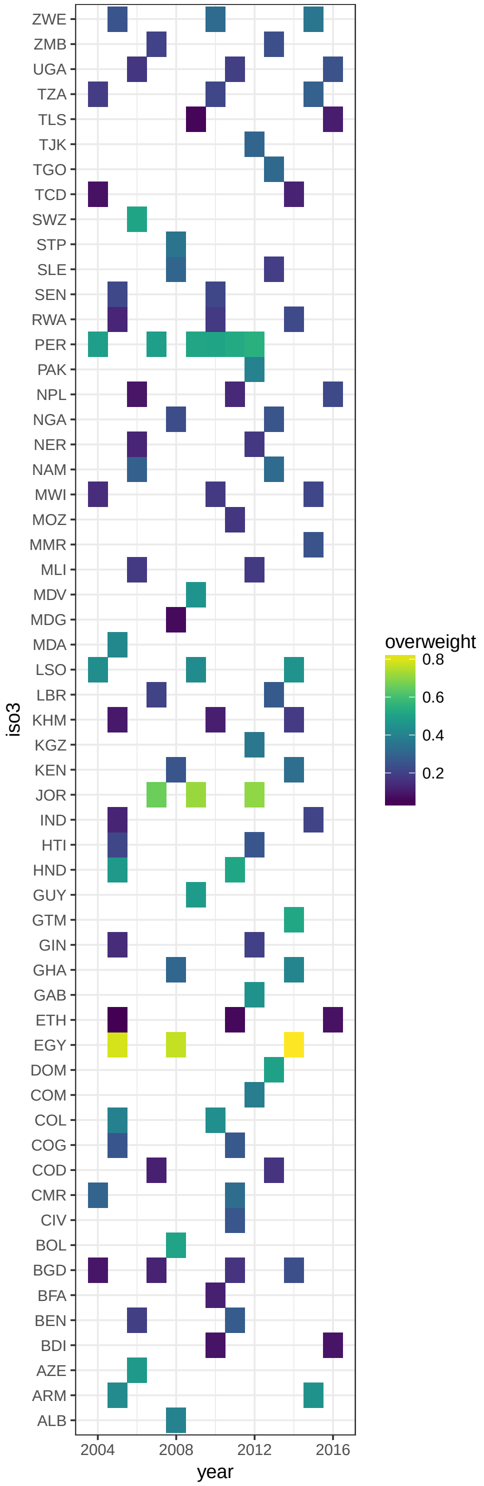

Figure B: Survey Coverage over Time and Countries for Demographic and Health Surveys. There is one survey in Morocco in 1995, and all World Health Surveys are representative of 2003.

3 Wealth Harmonization

In order to estimate the effect of wealth on obesity, we must standardize asset indicators found in the Demographic and Health Survey as well as the World Health Survey. We approach this problem with two methods: (1) the construction of a survey-specific wealth index and (2) the construction of a survey-standardized wealth index.

3.1 Survey-specific Wealth Index

To construct a survey-specific wealth index, we leverage the unique wealth assets available in each survey. From 1999 to present, the Demographic and Health Surveys contain a survey-specific wealth index is that is based on the first principal component of wealth assets relevant to standard of living within the country. Similarly, the World Health Surveys have survey-specific wealth questions meant to capture standard of living within the country. For example, while the survey for a low-income country may ask about electricity access, the survey in a high-income country does not. Unlike the Demographic and Health Survey, the World Health Survey does not provide a principal components analysis. So we preform a principal components analysis of all wealth questions in each World Health Survey (section 700) to produce an index. This measure captures the relative wealth of the individual with respect to others in the country during the survey-year.

3.2 Survey-standardized Wealth Index

To construct a survey-standardized wealth index, we identified the following variables present in most surveys back to 1995: water access, sanitation, floor material, electricity, radio, refrigerator, motorcycle, car, phone, rooms per person. We performed a principal components analysis of all of these variables jointly, across all survey-years and countries, in order to calculate the first principal component. Survey-standardized wealth index is restricted to the Demographic and Health Survey, because the World Health Survey asks different wealth questions in section 700 for high-income countries versus all other countries. High-income country surveys did not ask respondents about access to safe water, sanitation, electricity, or floor material.

4 Self-report bias

Multiple studies have shown that self-reported height can weight data can be biased. In women, weight data are typically under-reported, and in men, height and weight data are over-reported. The World Health Surveys are utilized in this study due to cross-country comparable data on both anthropometric measures and wealth assets. Four World Health Surveys were conducted via telephone: Australia, Israel, Luxembourg, and Norway. The rest were conducted in-person, where the survey proctor could see the person when reporting height and weight information. We consider bias in the data in two ways.

First, we examine the crude, national obesity rates of all World Health Surveys compared to the Global Burden of Disease national obesity estimates. The Global Burden of Disease estimates age-specific and all-age national obesity prevalence. As part of their modeling process, the Global Burden of Disease incorporates multiple data sources as well as a data-driven cross-walk to correct for self-report bias in surveys that use self-reported data. The results are in the below histogram, Figure C, which displays the distribution of the difference between these two sources. Negative values indicate that the World Health Surveys estimate a lower obesity rate that the Global Burden of Disease project. Positive differences indicate that the World Health Survey has a greater obesity rates than the estimated Global Burden of Disease obesity prevalence for that country. Slightly more World Health Surveys under-estimate obesity than over-estimate, but the on average the difference is centered at zero.

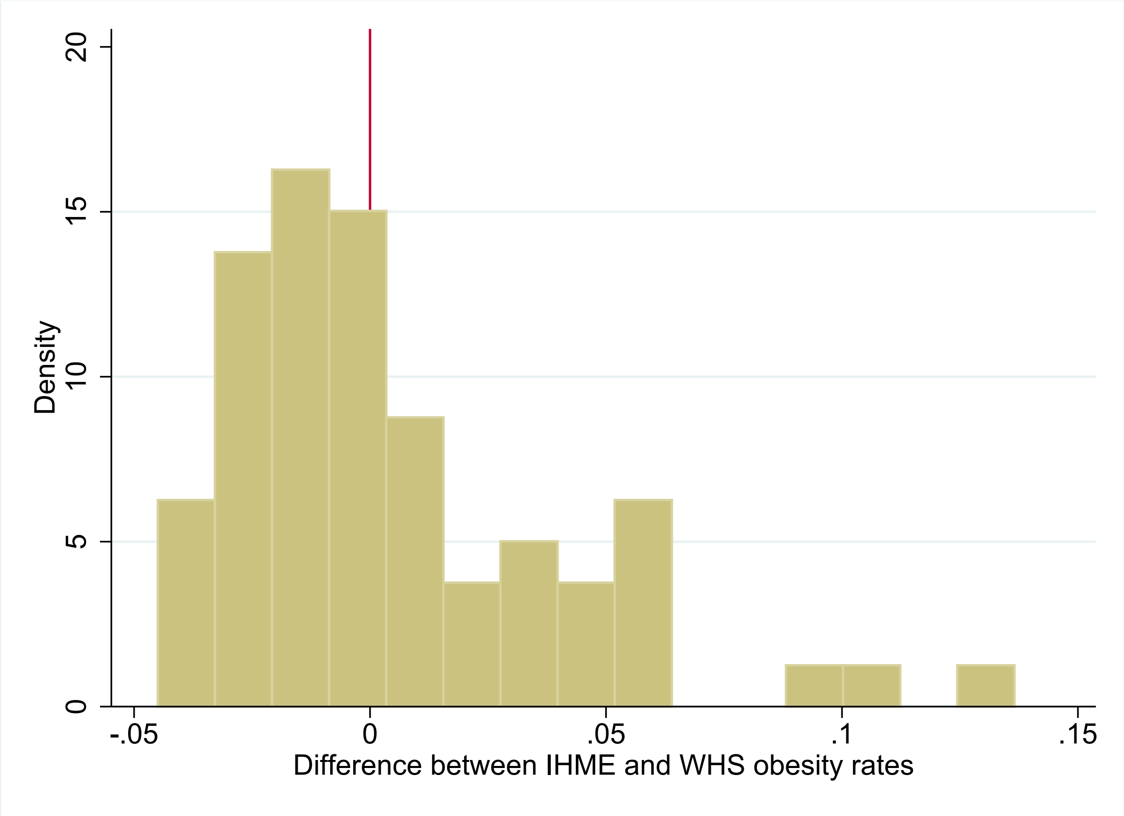
Density
Difference between IHME and WHS obesity rates
0
5
10
15
20
-.05
0
.05
.1
.15

Figure C

Second, we run a regression using Equation (1) in the manuscript, with an indicator for self-reported data, and replicate our analysis only using Demographic and Health Surveys. The results using the self-report indicator for the outcomes of BMI and obesity are reported in Section 5, in Tables 5 and 9. Table 5 indicates that individuals self-reporting their weight are less likely to report a BMI that classifies as obese, which is significant at the 1% level. Table 9 indicates that self-reported BMIs are on average 0.015 units lower, which is significant at the 1% level. Thus controlling for other factors, self-reporting the data does result in a downward bias. However, replication of the regression analysis in the Demographic and Health Surveys, which only include anthropometric data, is much more encouraging. The results are in Table 7 (column 3), Table 8 (column 4), Table 9 (column 3), Table 10 (column 3), Table 11 (column 3), and Table 12 (column 4). We see that the coefficient direction and magnitude remain very similar.

5 Statistical Analysis

This section presents regression tables underlying Figures 2, 3, and 4 of the main text for each of three outcome variables: BMI, a binary indicator for obesity, and a binary indicator for overweight. Below are descriptions of the Tables G - L included in this section.

Table G: This table presents our regression results using individual obesity as the outcome variable. Because the outcome is a binary variable, we use logistic regression, with country and year fixed effects. Column (1) displays the results using the full data set and survey-specific personal wealth deciles. The reference group is the wealthiest decile. Column (2) displays the results using just the Demographic and Health Surveys and our survey-standardized wealth index. Column (3) displays the results using just the Demographic and Health Surveys and survey-specific personal wealth deciles. The reference group is the wealthiest decile.

Table H: This table continues using a binary obesity variable and logistic regression. Column (1) mirrors Table G, column 1 and additionally includes 3 indicator variables for educational attainment. Column (2) mirrors Table G, column 1 and additionally includes an indicator for self-reported data. Column (3) mirrors Table G, column 2 and additionally includes 3 indicator variables for educational attainment. Column (4) mirrors Table G, column 3 and additionally includes 3 indicator variables for educational attainment.

Table I: Follows the same structure as Table G, but now uses a binary overweight outcome variable.

Table J: Follows the same structure as Table H, using a binary overweight outcome variable.

Table K: Follows the same structure as Table G, but now using linear regression and $\log(BMI)$ as the outcome variable.

Table L: Follows the same structure as Table H, using linear regression and $\log(BMI)$ as the outcome variable.

Table G: Obese

	Dependent variable:		
	obese		
	(1)	(2)	(3)
log(GDP per capita)	-0.875^{***}	-0.300^{*}	-1.088^{***}
	(0.049)	(0.161)	(0.159)
Wealth Decile 1	-9.616^{***}		-15.473^{***}
	(0.174)		(0.445)
Wealth Decile 2	-9.724^{***}		-14.360^{***}
	(0.164)		(0.377)
Wealth Decile 3	-9.203^{***}		-13.071^{***}
	(0.154)		(0.325)
Wealth Decile 4	-8.746^{***}		-12.948^{***}
	(0.146)		(0.301)
Wealth Decile 5	-7.641^{***}		-11.644^{***}
	(0.136)		(0.273)
Wealth Decile 6	-6.793^{***}		-9.839^{***}
	(0.129)		(0.247)
Wealth Decile 7	-5.334^{***}		-8.168^{***}
	(0.121)		(0.230)
Wealth Decile 8	-3.943^{***}		-6.181^{***}
	(0.115)		(0.215)
Wealth Decile 9	-1.883^{***}		-3.392^{***}
	(0.108)		(0.203)
Survey-standardized Wealth		1.442^{***}	
		(0.037)	
Age	0.060^{***}	0.059^{***}	0.061^{***}
	(0.0002)	(0.001)	(0.001)
Female	-0.225^{***}		
	(0.016)		
log(GDP per capita):Wealth Decile 1	0.946^{***}		1.602^{***}
	(0.020)		(0.050)
log(GDP per capita):Wealth Decile 2	0.985^{***}		1.516^{***}
	(0.019)		(0.043)
log(GDP per capita):Wealth Decile 3	0.949^{***}		1.404^{***}
	(0.018)		(0.037)
log(GDP per capita):Wealth Decile 4	0.917^{***}		1.418^{***}
	(0.017)		(0.034)
log(GDP per capita):Wealth Decile 5	0.808^{***}		1.288^{***}
	(0.016)		(0.031)
log(GDP per capita):Wealth Decile 6	0.729^{***}		1.101^{***}
	(0.015)		(0.029)
log(GDP per capita):Wealth Decile 7	0.576^{***}		0.918^{***}
	(0.014)		(0.027)
log(GDP per capita):Wealth Decile 8	0.434^{***}		0.701^{***}
	(0.013)		(0.025)
log(GDP per capita):Wealth Decile 9	0.211^{***}		0.389^{***}
	(0.013)		(0.024)
log(GDP per capita):wealth		-0.138^{***}	
		(0.004)	
Constant	4.208^{***}	-1.634	6.277^{***}
	(0.430)	(1.362)	(1.350)
Countries	Yes	Yes	Yes
Years	Yes	Yes	Yes
Observations	2,243,991	388,609	388,609
Log Likelihood	-529,499.000	-134,776.000	-132,355.000
Akaike Inf. Crit.	1,059,274.000	269,652.000	264,842.000
Note:			*p<0.1; **p<0.05; ***p<0.01

Table H: Obese

	Dependent variable:			
	obese			
	(1)	(2)	(3)	(4)
log(GDP per capita)	-0.853^{***}	-0.796^{***}	-0.223	-1.023^{***}
	(0.049)	(0.050)	(0.161)	(0.160)
Wealth Decile 1	-9.010^{***}	-9.587^{***}		-14.758^{***}
	(0.173)	(0.174)		(0.447)
Wealth Decile 2	-9.204^{***}	-9.771^{***}		-13.726^{***}
	(0.164)	(0.164)		(0.379)
Wealth Decile 3	-8.743^{***}	-9.308^{***}		-12.466^{***}
	(0.154)	(0.154)		(0.327)
Wealth Decile 4	-8.346^{***}	-8.844^{***}		-12.407^{***}
	(0.146)	(0.145)		(0.303)
Wealth Decile 5	-7.283^{***}	-7.748^{***}		-11.161^{***}
	(0.137)	(0.136)		(0.275)
Wealth Decile 6	-6.491^{***}	-6.904^{***}		-9.441^{***}
	(0.130)	(0.129)		(0.249)
Wealth Decile 7	-5.096^{***}	-5.420^{***}		-7.840^{***}
	(0.122)	(0.121)		(0.231)
Wealth Decile 8	-3.770^{***}	-4.019^{***}		-5.954^{***}
	(0.115)	(0.115)		(0.216)
Wealth Decile 9	-1.808^{***}	-1.953^{***}		-3.282^{***}
	(0.108)	(0.108)		(0.204)
Survey-standardized Wealth			1.310^{***}	
			(0.037)	
Primary School	0.349^{***}		0.416^{***}	0.204^{***}
	(0.009)		(0.020)	(0.020)
Secondary Education	0.315^{***}		0.526^{***}	0.174^{***}
	(0.009)		(0.020)	(0.020)
Tertiary Education	0.112^{***}		0.235^{***}	-0.116^{***}
	(0.012)		(0.023)	(0.024)
Age	0.062^{***}	0.061^{***}	0.062^{***}	0.061^{***}
	(0.0003)	(0.0003)	(0.001)	(0.001)
Female	-0.240^{***}	-0.419^{***}		
	(0.016)	(0.018)		
Self-Reported		-0.960^{***}		
		(0.030)		
log(GDP per capita):Wealth Decile 1	0.880^{***}	0.943^{***}		1.507^{***}
	(0.020)	(0.020)		(0.050)
log(GDP per capita):Wealth Decile 2	0.926^{***}	0.990^{***}		1.429^{***}
	(0.019)	(0.019)		(0.043)
log(GDP per capita):Wealth Decile 3	0.895^{***}	0.960^{***}		1.321^{***}
	(0.018)	(0.018)		(0.037)
log(GDP per capita):Wealth Decile 4	0.869^{***}	0.928^{***}		1.342^{***}
	(0.017)	(0.017)		(0.035)
log(GDP per capita):Wealth Decile 5	0.765^{***}	0.820^{***}		1.220^{***}
	(0.016)	(0.016)		(0.032)
log(GDP per capita):Wealth Decile 6	0.691^{***}	0.741^{***}		1.043^{***}
	(0.015)	(0.015)		(0.029)
log(GDP per capita):Wealth Decile 7	0.546^{***}	0.586^{***}		0.870^{***}
	(0.014)	(0.014)		(0.027)
log(GDP per capita):Wealth Decile 8	0.411^{***}	0.442^{***}		0.666^{***}
	(0.013)	(0.013)		(0.025)
log(GDP per capita):Wealth Decile 9	0.199^{***}	0.218^{***}		0.370^{***}
	(0.013)	(0.013)		(0.024)
log(GDP per capita):wealth			-0.123^{***}	
			(0.004)	
Constant	3.579^{***}	3.945^{***}	-2.761^{**}	5.748^{***}
	(0.431)	(0.433)	(1.363)	(1.351)
Countries	Yes	Yes	Yes	Yes
Years	Yes	Yes	Yes	Yes
Observations	2,243,991	2,243,991	388,609	388,609
Log Likelihood	-528,403.000	-528,964.000	-134,267.000	-132,101.000
Akaike Inf. Crit.	1,057,088.000	1,058,206.000	268,640.000	264,340.000
Note:				*p<0.1; **p<0.05; ***p<0.01

Table I: Overweight

	Dependent variable:		
	overweight		
	(1)	(2)	(3)
log(GDP per capita)	-0.410^{***}	-0.179^{*}	-0.768^{***}
	(0.029)	(0.097)	(0.099)
Wealth Decile 1	-6.710^{***}		-10.436^{***}
	(0.095)		(0.207)
Wealth Decile 2	-6.772^{***}		-10.154^{***}
	(0.093)		(0.199)
Wealth Decile 3	-6.711^{***}		-10.518^{***}
	(0.091)		(0.197)
Wealth Decile 4	-6.369^{***}		-10.437^{***}
	(0.088)		(0.191)
Wealth Decile 5	-5.797^{***}		-9.148^{***}
	(0.086)		(0.183)
Wealth Decile 6	-5.086^{***}		-8.184^{***}
	(0.083)		(0.178)
Wealth Decile 7	-4.124^{***}		-6.886^{***}
	(0.080)		(0.173)
Wealth Decile 8	-2.923^{***}		-5.339^{***}
	(0.078)		(0.170)
Wealth Decile 9	-1.341^{***}		-2.909^{***}
	(0.077)		(0.168)
Survey-standardized Wealth		1.263^{***}	
		(0.025)	
Age	0.060^{***}	0.052^{***}	0.053^{***}
	(0.0002)	(0.0004)	(0.0004)
Female	0.100^{***}		
	(0.011)		
log(GDP per capita):Wealth Decile 1	0.615^{***}		1.040^{***}
	(0.011)		(0.024)
log(GDP per capita):Wealth Decile 2	0.646^{***}		1.040^{***}
	(0.011)		(0.023)
log(GDP per capita):Wealth Decile 3	0.661^{***}		1.106^{***}
	(0.011)		(0.023)
log(GDP per capita):Wealth Decile 4	0.641^{***}		1.124^{***}
	(0.010)		(0.023)
log(GDP per capita):Wealth Decile 5	0.592^{***}		0.992^{***}
	(0.010)		(0.022)
log(GDP per capita):Wealth Decile 6	0.528^{***}		0.894^{***}
	(0.010)		(0.021)
log(GDP per capita):Wealth Decile 7	0.435^{***}		0.760^{***}
	(0.010)		(0.021)
log(GDP per capita):Wealth Decile 8	0.313^{***}		0.594^{***}
	(0.009)		(0.020)
log(GDP per capita):Wealth Decile 9	0.145^{***}		0.322^{***}
	(0.009)		(0.020)
log(GDP per capita):wealth		-0.120^{***}	
		(0.003)	
Constant	1.596^{***}	-0.863	5.384^{***}
	(0.252)	(0.821)	(0.838)
Countries	Yes	Yes	Yes
Year	Yes	Yes	Yes
Observations	2,243,991	388,609	388,609
Log Likelihood	-1,069,654.000	-216,965.000	-212,449.000
Akaike Inf. Crit.	2,139,583.000	434,030.000	425,029.000
Note:			*p$<$0.1; **p$<$0.05; ***p$<$0.01

Table J: Overweight

	Dependent variable:		
	overweight		
	(1)	(2)	(3)
log(GDP per capita)	-0.372^{***}	-0.104	-0.673^{***}
	(0.029)	(0.097)	(0.099)
Wealth Decile 1	-6.112^{***}		-9.366^{***}
	(0.095)		(0.209)
Wealth Decile 2	-6.222^{***}		-9.184^{***}
	(0.093)		(0.201)
Wealth Decile 3	-6.209^{***}		-9.602^{***}
	(0.091)		(0.199)
Wealth Decile 4	-5.921^{***}		-9.607^{***}
	(0.088)		(0.193)
Wealth Decile 5	-5.394^{***}		-8.411^{***}
	(0.086)		(0.185)
Wealth Decile 6	-4.739^{***}		-7.555^{***}
	(0.083)		(0.180)
Wealth Decile 7	-3.846^{***}		-6.367^{***}
	(0.081)		(0.175)
Wealth Decile 8	-2.724^{***}		-4.987^{***}
	(0.079)		(0.171)
Wealth Decile 9	-1.253^{***}		-2.726^{***}
	(0.077)		(0.168)
Survey-standardized Wealth		1.107^{***}	
		(0.026)	
Primary School	0.408^{***}	0.510^{***}	0.325^{***}
	(0.006)	(0.013)	(0.013)
Secondary Education	0.403^{***}	0.695^{***}	0.337^{***}
	(0.006)	(0.014)	(0.014)
Tertiary Education	0.310^{***}	0.468^{***}	0.044^{**}
	(0.007)	(0.016)	(0.018)
Age	0.063^{***}	0.056^{***}	0.055^{***}
	(0.0002)	(0.0004)	(0.0005)
Female	0.084^{***}		
	(0.011)		
log(GDP per capita):Wealth Decile 1	0.559^{***}		0.910^{***}
	(0.011)		(0.024)
log(GDP per capita):Wealth Decile 2	0.592^{***}		0.920^{***}
	(0.011)		(0.024)
log(GDP per capita):Wealth Decile 3	0.610^{***}		0.991^{***}
	(0.011)		(0.023)
log(GDP per capita):Wealth Decile 4	0.595^{***}		1.019^{***}
	(0.010)		(0.023)
log(GDP per capita):Wealth Decile 5	0.550^{***}		0.898^{***}
	(0.010)		(0.022)
log(GDP per capita):Wealth Decile 6	0.491^{***}		0.812^{***}
	(0.010)		(0.021)
log(GDP per capita):Wealth Decile 7	0.404^{***}		0.692^{***}
	(0.010)		(0.021)
log(GDP per capita):Wealth Decile 8	0.291^{***}		0.547^{***}
	(0.009)		(0.020)
log(GDP per capita):Wealth Decile 9	0.135^{***}		0.296^{***}
	(0.009)		(0.020)
log(GDP per capita):wealth		-0.104^{***}	
		(0.003)	
Constant	0.562^{**}	-2.195^{***}	4.341^{***}
	(0.252)	(0.823)	(0.840)
Countries	Yes	Yes	Yes
Year	Yes	Yes	Yes
Observations	2,243,991	388,609	388,609
Log Likelihood	-1,066,418.000	-215,587.000	-211,849.000
Akaike Inf. Crit.	2,133,117.000	431,281.000	423,836.000
Note:			*p<0.1; **p<0.05; ***p<0.01

Table K: BMI

	Dependent variable:		
	log(bmi)		
	(1)	(2)	(3)
log(GDP per capita)	-0.022^{***}	0.005	-0.044^{***}
	(0.002)	(0.006)	(0.006)
Wealth Decile 1	-0.369^{***}		-0.760^{***}
	(0.006)		(0.012)
Wealth Decile 2	-0.381^{***}		-0.755^{***}
	(0.006)		(0.013)
Wealth Decile 3	-0.405^{***}		-0.799^{***}
	(0.006)		(0.013)
Wealth Decile 4	-0.408^{***}		-0.843^{***}
	(0.006)		(0.013)
Wealth Decile 5	-0.390^{***}		-0.781^{***}
	(0.006)		(0.013)
Wealth Decile 6	-0.371^{***}		-0.734^{***}
	(0.006)		(0.013)
Wealth Decile 7	-0.313^{***}		-0.642^{***}
	(0.006)		(0.013)
Wealth Decile 8	-0.240^{***}		-0.511^{***}
	(0.006)		(0.012)
Wealth Decile 9	-0.119^{***}		-0.306^{***}
	(0.006)		(0.013)
Survey-standardized Wealth		0.096^{***}	
		(0.002)	
Age	0.005^{***}	0.004^{***}	0.004^{***}
	(0.00001)	(0.00003)	(0.00003)
Female	0.007^{***}		
	(0.001)		
log(GDP per capita):Wealth Decile 1	0.029^{***}		0.074^{***}
	(0.001)		(0.001)
log(GDP per capita):Wealth Decile 2	0.032^{***}		0.076^{***}
	(0.001)		(0.002)
log(GDP per capita):Wealth Decile 3	0.037^{***}		0.083^{***}
	(0.001)		(0.002)
log(GDP per capita):Wealth Decile 4	0.039^{***}		0.090^{***}
	(0.001)		(0.002)
log(GDP per capita):Wealth Decile 5	0.038^{***}		0.084^{***}
	(0.001)		(0.002)
log(GDP per capita):Wealth Decile 6	0.037^{***}		0.080^{***}
	(0.001)		(0.001)
log(GDP per capita):Wealth Decile 7	0.032^{***}		0.071^{***}
	(0.001)		(0.002)
log(GDP per capita):Wealth Decile 8	0.025^{***}		0.057^{***}
	(0.001)		(0.001)
log(GDP per capita):Wealth Decile 9	0.012^{***}		0.034^{***}
	(0.001)		(0.002)
log(GDP per capita):wealth		-0.009^{***}	
		(0.0002)	
Constant	3.292^{***}	2.998^{***}	3.528^{***}
	(0.015)	(0.054)	(0.053)
Countries	Yes	Yes	Yes
Year	Yes	Yes	Yes
Observations	2,243,991	388,609	388,609
Log Likelihood	867,384.000	146,718.000	152,333.000
Akaike Inf. Crit.	-1,734,492.000	-293,335.000	-304,535.000
Note:			*p<0.1; **p<0.05; ***p<0.01

Table L: BMI

	Dependent variable:			
	log(bmi)			
	(1)	(2)	(3)	(4)
log(GDP per capita)	-0.020^{***} (0.002)	-0.023^{***} (0.002)	0.008 (0.006)	-0.037^{***} (0.006)
Wealth Decile 1	-0.334^{***} (0.006)	-0.369^{***} (0.006)		-0.673^{***} (0.013)
Wealth Decile 2	-0.346^{***} (0.006)	-0.383^{***} (0.006)		-0.676^{***} (0.013)
Wealth Decile 3	-0.371^{***} (0.006)	-0.407^{***} (0.006)		-0.723^{***} (0.013)
Wealth Decile 4	-0.377^{***} (0.006)	-0.410^{***} (0.006)		-0.773^{***} (0.013)
Wealth Decile 5	-0.360^{***} (0.006)	-0.391^{***} (0.006)		-0.719^{***} (0.013)
Wealth Decile 6	-0.346^{***} (0.006)	-0.373^{***} (0.006)		-0.681^{***} (0.013)
Wealth Decile 7	-0.292^{***} (0.006)	-0.314^{***} (0.006)		-0.599^{***} (0.013)
Wealth Decile 8	-0.226^{***} (0.006)	-0.241^{***} (0.006)		-0.481^{***} (0.012)
Wealth Decile 9	-0.113^{***} (0.006)	-0.120^{***} (0.006)		-0.291^{***} (0.013)
Survey-standardized Wealth			0.081^{***} (0.002)	
Primary School	0.032^{***} (0.0003)		0.044^{***} (0.001)	0.031^{***} (0.001)
Secondary Education	0.031^{***} (0.0003)		0.058^{***} (0.001)	0.030^{***} (0.001)
Tertiary Education	0.026^{***} (0.0005)		0.040^{***} (0.001)	0.005^{***} (0.001)
Self-Reported		-0.015^{***} (0.001)		
Age	0.005^{***} (0.00001)	0.005^{***} (0.00001)	0.005^{***} (0.00003)	0.004^{***} (0.00003)
Female	0.006^{***} (0.001)	0.003^{***} (0.001)		
log(GDP per capita):Wealth Decile 1	0.026^{***} (0.001)	0.029^{***} (0.001)		0.063^{***} (0.002)
log(GDP per capita):Wealth Decile 2	0.029^{***} (0.001)	0.033^{***} (0.001)		0.066^{***} (0.002)
log(GDP per capita):Wealth Decile 3	0.034^{***} (0.001)	0.037^{***} (0.001)		0.073^{***} (0.002)
log(GDP per capita):Wealth Decile 4	0.036^{***} (0.001)	0.039^{***} (0.001)		0.081^{***} (0.002)
log(GDP per capita):Wealth Decile 5	0.035^{***} (0.001)	0.038^{***} (0.001)		0.076^{***} (0.002)
log(GDP per capita):Wealth Decile 6	0.035^{***} (0.001)	0.037^{***} (0.001)		0.073^{***} (0.002)
log(GDP per capita):Wealth Decile 7	0.030^{***} (0.001)	0.032^{***} (0.001)		0.065^{***} (0.002)
log(GDP per capita):Wealth Decile 8	0.024^{***} (0.001)	0.025^{***} (0.001)		0.053^{***} (0.001)
log(GDP per capita):Wealth Decile 9	0.012^{***} (0.001)	0.013^{***} (0.001)		0.032^{***} (0.002)
log(GDP per capita):wealth			-0.007^{***} (0.0002)	
Constant	3.214^{***} (0.015)	3.306^{***} (0.015)	2.915^{***} (0.053)	3.444^{***} (0.053)
Countries	Yes	Yes	Yes	Yes
Year	Yes	Yes	Yes	Yes
Observations	2,243,991	2,243,991	388,609	388,609
Log Likelihood	872,602.000	867,470.000	148,801.000	153,362.000
Akaike Inf. Crit.	-1,744,923.000	-1,734,662.000	-297,495.000	-306,587.000
Note:				*p<0.1; **p<0.05; ***p<0.01

6 Robustness Checks

In this section, we examine how sensitive our main result – the existence of a transition of overweight and obesity to being relatively concentrated amongst the rich to the poor – is to only using Demographic and Health Surveys (Figure D); using country-specific age and sex trends (Figure E); and using wealth quintiles as opposed to wealth deciles (Figure F). These figures are presented in the following pages.

For Figure D, the result does not change. This is unsurprising, as most of our data in the main analysis is from the Demographic and Health surveys. There is more uncertainty in the higher GDP per capita ranges, which also is to be expected as the Demographic and Health Surveys only capture low- and middle- income countries.

Using country-specific age and sex trends, we see that the effect of wealth slightly attenuates, suggesting that country-specific age and sex trends were slightly biasing the the wealth effects. This specification, however, does not change that there is a clear gradient reversal across the GDP per capita range.

Finally, We see that using wealth quintiles also exhibits a similar trend as our main results with wealth deciles.

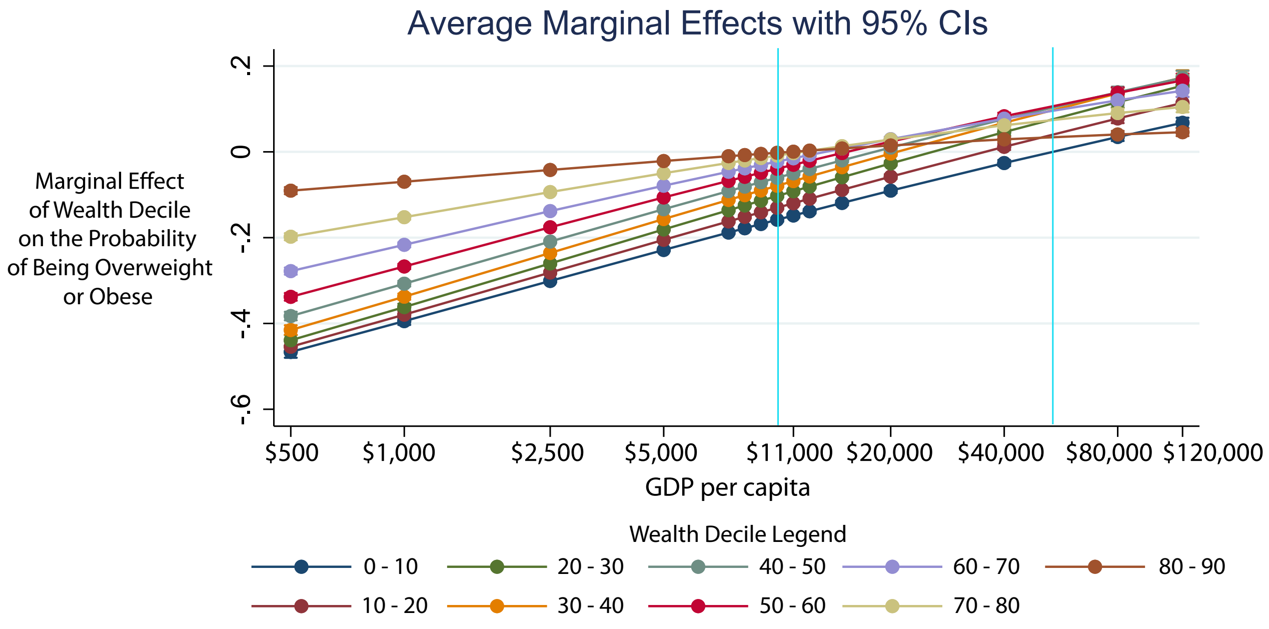

Figure D: Only Demographic and Health Surveys

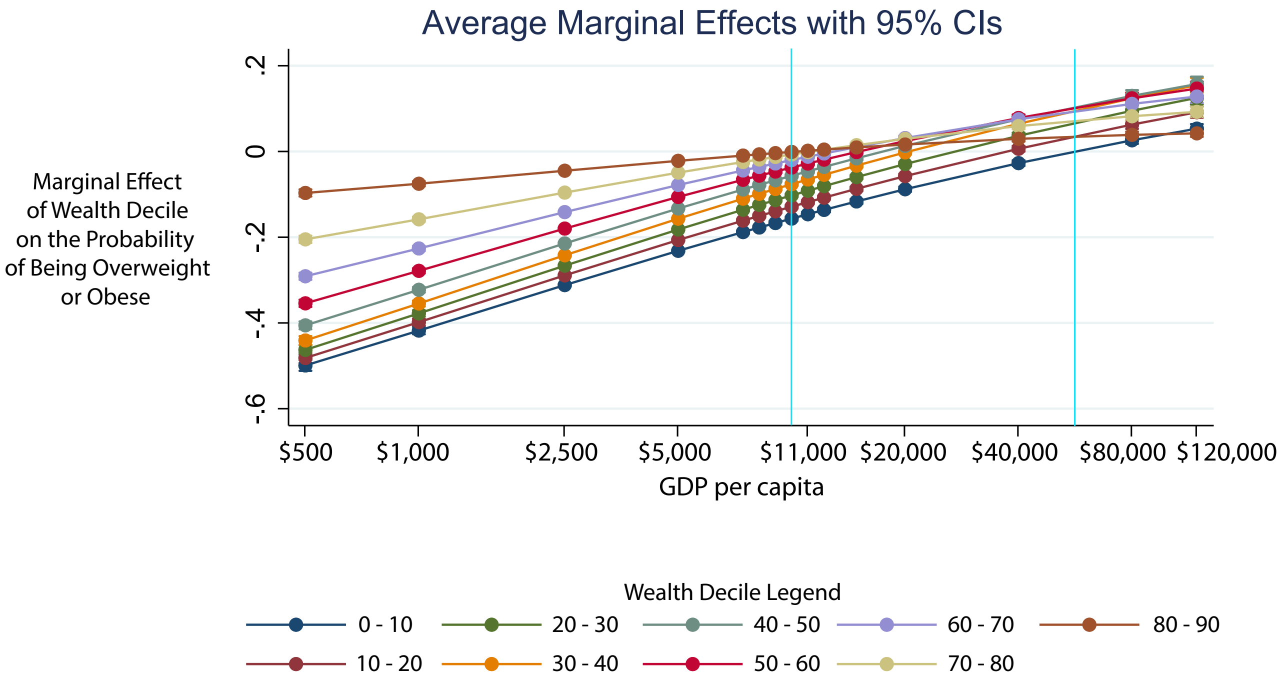

Figure E: Country-specific age and sex trends

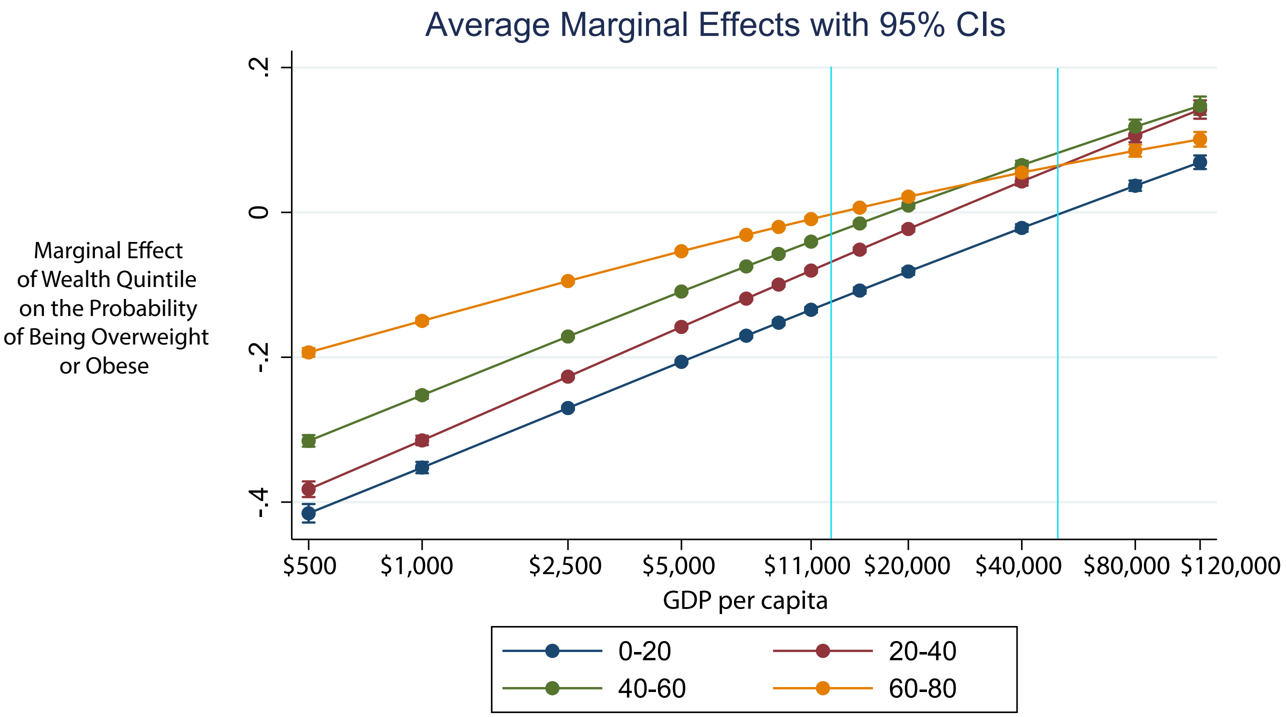

Figure F: Wealth Quintiles

7 Meta-regression

As an alternate modeling strategy to fixed effects regression, we employ a meta-analytic technique to compare within country wealth gradients across GDP per capita. We implemented this methodology and found that our results still hold, using both an absolute measure of wealth (using just the Demographic and Health Survey sample) and a relative measure of wealth (using the Demographic and Health Surveys and World Health Surveys).

For absolute wealth, the regression equations that we estimated were, separately for each survey, for individual i:

Equation A:

$$outcome_i = \alpha + \beta wealth_i + \delta age_i + \gamma sex_i + \theta education_i + \epsilon_i$$

For the absolute wealth measure, this produces 182 β coefficients which then enter the following equation, for country c:

Equation B:

$$\beta_c = \xi + \pi \log\left(GDPpc_c\right) + \nu_c$$

For relative wealth, the regression equations that we estimated were, separately for each survey, for individual i:

Equation C:

$$outcome_i = \alpha + \beta_1 1(wealth_i = Poorest) + \beta_2 1(wealth_i = Poorer) + \beta_3 1(wealth_i = Middle)$$

$$+\beta_4 1(wealth_i = Richer) + \delta age_i + \gamma sex_i + \theta education_i + \epsilon_i$$

The richest wealth group is the reference category, and 1() denotes a 0/1 variable that indicates to which wealth group an individual belongs. For the relative wealth measure, this produces 784 β coefficients which then enter the following equation, separately for each wealth quintile w, for country c:

Equation D:

$$\beta_{cw} = \xi + \pi \log\left(GDPpc_c\right) + \nu_c$$

To display our results, we take two approaches. First, using the Equations A and C on this page, we graph the β coefficients as a function of GDP per capita. This is meant to give a sense of how the within-country estimate of the effect of wealth on BMI, overweight, and obesity, varies with GDP per capita. Each dot represents a coefficient from a within country regression. We fit a lowess curve through these dots to show how the coefficients on wealth vary with GDP per capita. The vertical lines show where the point estimates become positive. Note that the shaded confidence intervals can give a sense of the variance, but do not incorporate the sample size or standard errors of the point estimates, as we do in the second approach (Described after the graphs).

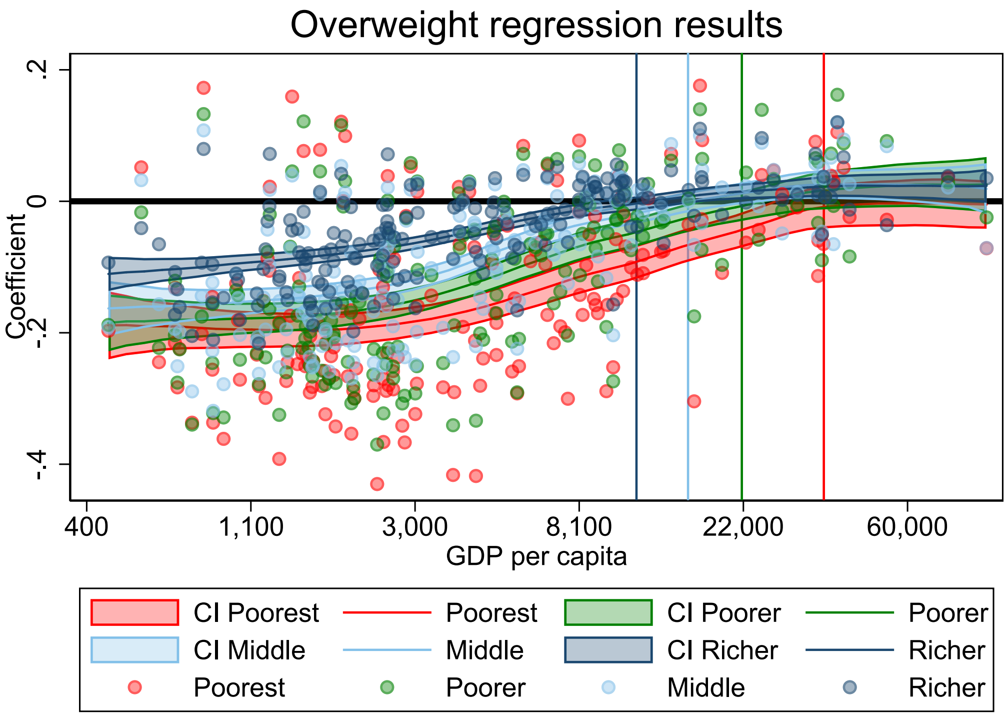

Figure G: Effect of Wealth Quintile on Overweight, across GDP per capita

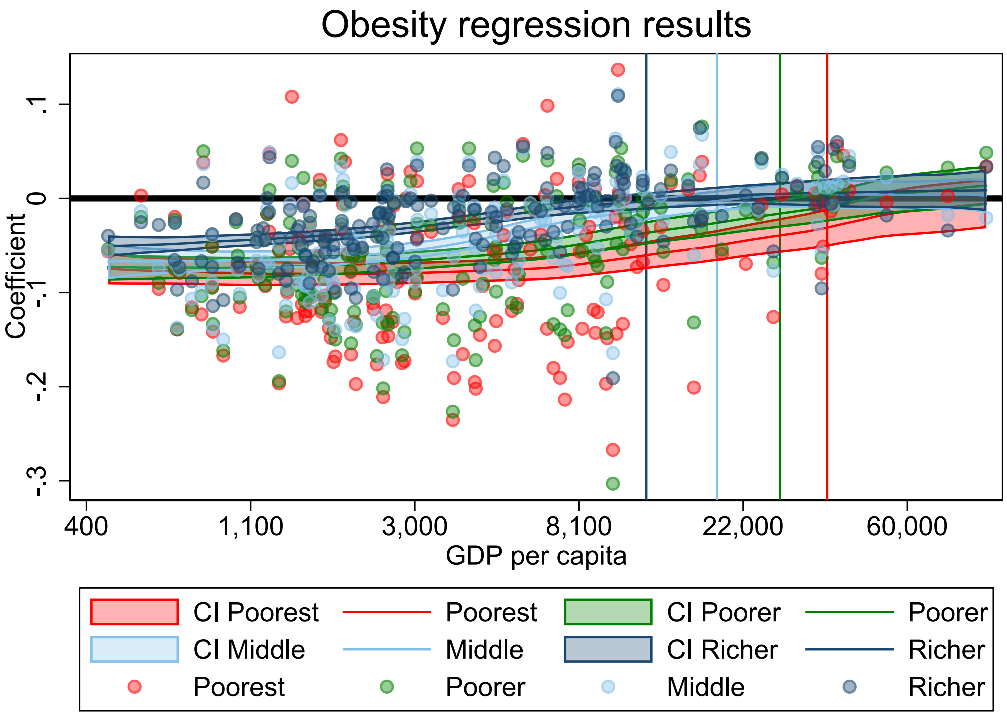

Figure H: Effect of Wealth Quintile on Obesity, across GDP per capita

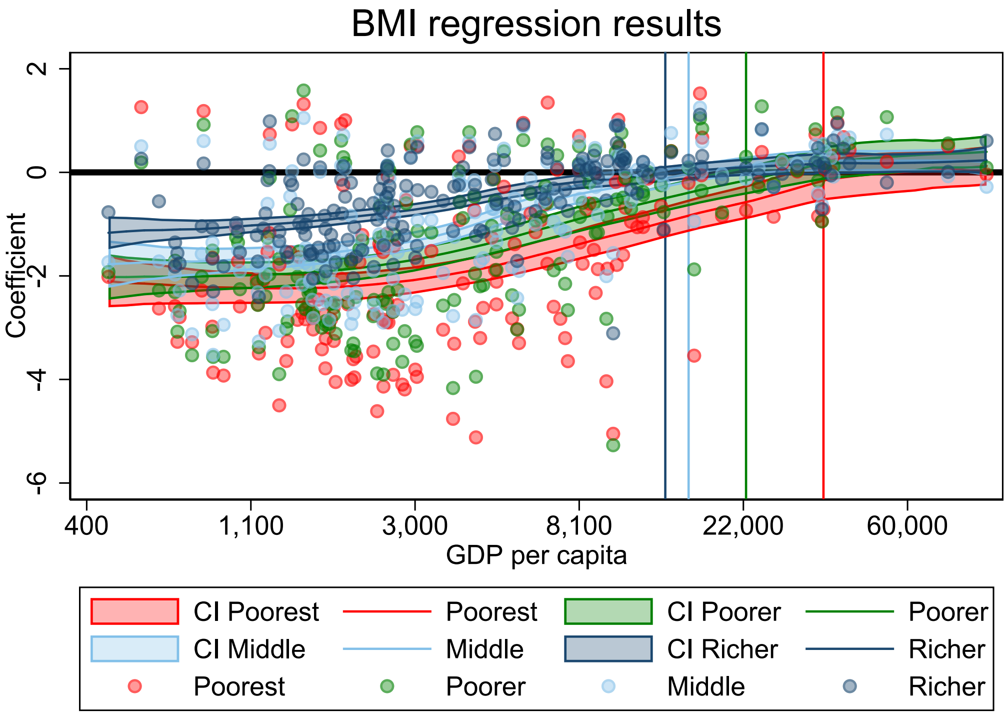

Figure I: Effect of Wealth Quintile on BMI, across GDP per capita

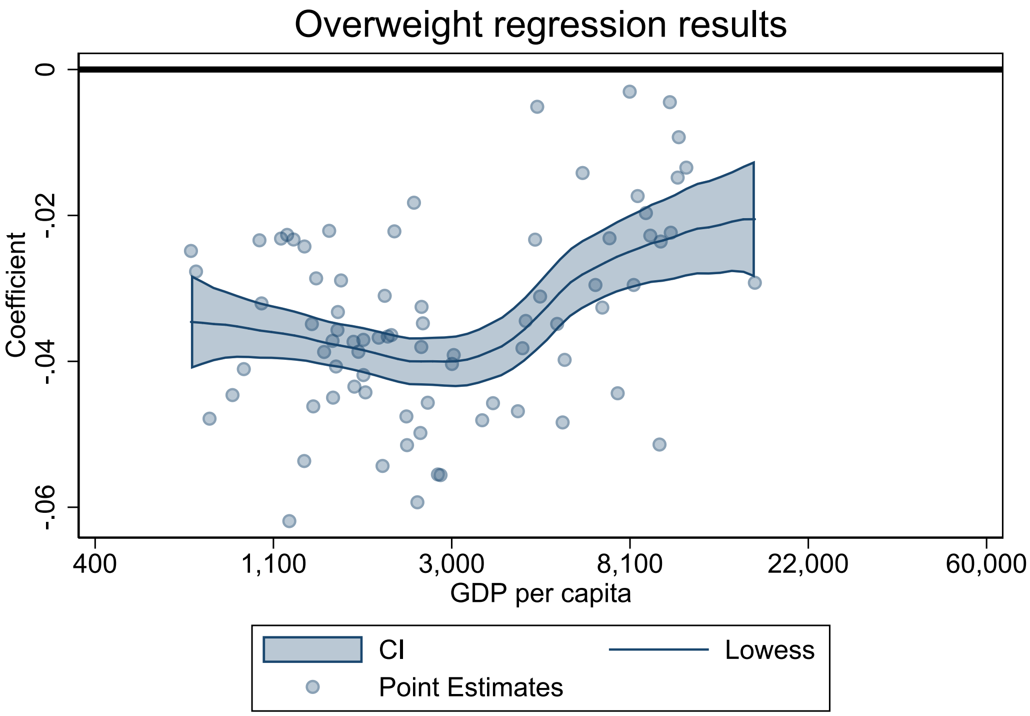

Figure J: Effect ofDHS Absolute Wealth on Overweight, across GDP per capita

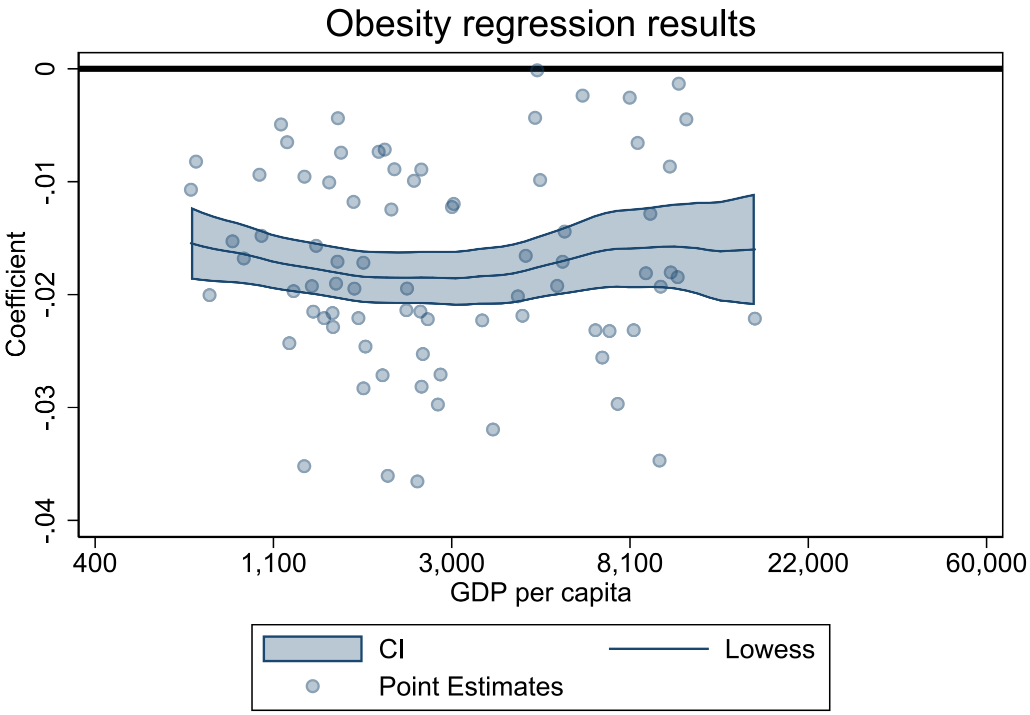

Figure K: Effect of DHS Absolute Wealth on Obesity, across GDP per capita

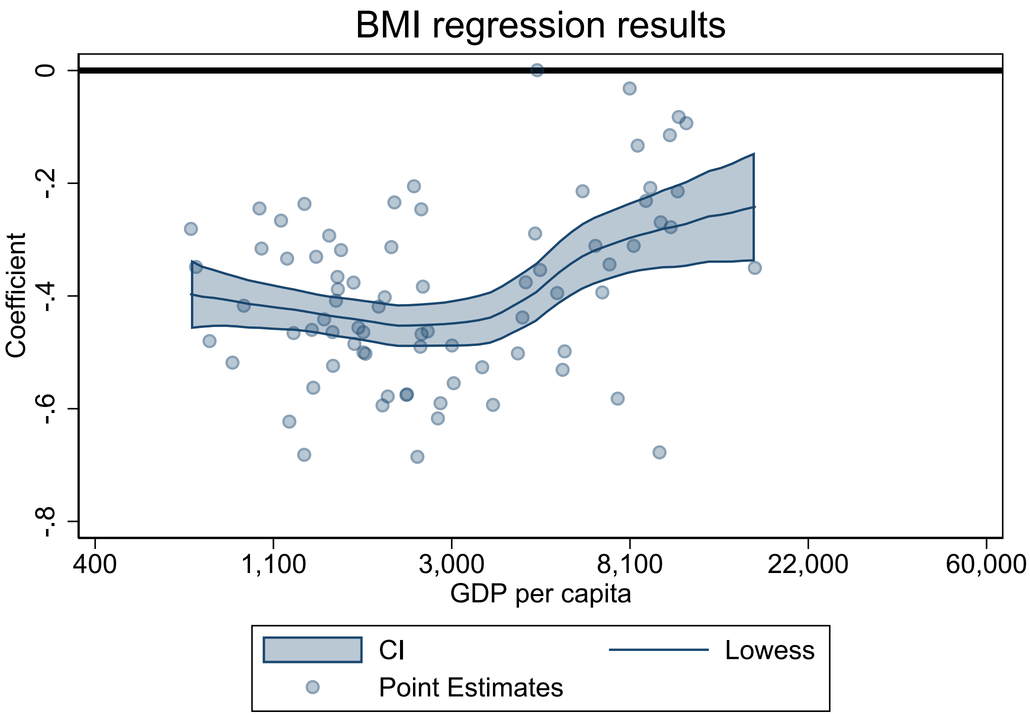

Figure L: Effect of DHS Absolute Wealth on BMI, across GDP per capita

Second, we report the meta-regression results of Equations B and D. We use the R package metafor to perform the meta-regression analysis. Some notation for the statistics reported in the table:

τ^2: Estimated amount of residual heterogeneity

τ: square root of estimated τ^2 value

I^2: residual heterogeneity / unaccounted variability

H^2: unaccounted variability / sampling variability

R^2: amount of heterogeneity accounted for

Now the results for wealth quintiles.

Table N: Effect of DHS Absolute Wealth on BMI, across GDP per capita

	Estimate	SE	Z-value	p-value	Lower CI	Upper CI
Intercept	-0.9093	0.1671	-5.4419	<.0001	-1.2369	-0.5818
log(GDPpc)	0.0650	0.0209	3.1081	0.0019	0.0240	0.1059
τ^2	0.0203					
τ	0.1425					
I^2	98.00%					
H^2	49.94					
R^2	10.66%					

Table O: Effect of DHS Absolute Wealth on Overweight, across GDP per capita

	Estimate	SE	Z-value	p-value	Lower CI	Upper CI
Intercept	-0.0814	0.0140	-5.8275	<.0001	-0.1088	-0.0541
log(GDPpc)	0.0060	0.0017	3.4104	0.0006	0.0025	0.0094
τ^2	0.0001					
τ	0.0118					
I^2	97.26%					
H^2	36.45					
R^2	12.99%					

Table P: Effect of DHS Absolute Wealth on Obesity, across GDP per capita

	Estimate	SE	Z-value	p-value	Lower CI	Upper CI
Intercept	-0.0175	0.0100	-1.7541	0.0794	-0.0370	0.0021
log(GDPpc)	0.0001	0.0012	0.0431	0.9657	-0.0024	0.0025
τ^2	0.0001					
τ	0.0084					
I^2	97.97%					
H^2	49.27					
R^2	0.00%					

Table Q: Effect of Wealth Quintiles on Overweight, across GDP per capita

	Estimate	SE	Z-value	p-value	Lower CI	Upper CI
Intercept for Poorest	-0.6090	0.0655	-9.2945	<.0001	-0.7374	-0.4806
log(GDPpc) for Poorest	0.0557	0.0078	7.1109	<.0001	0.0404	0.0711
R^2 for Poorest	22.57%					
Intercept for Poorer	-0.6514	0.0558	-11.6643	<.0001	-0.7609	-0.5420
log(GDPpc) for Poorer	0.0646	0.0067	9.6508	<.0001	0.0514	0.0777
R^2 for Poorer	35.72%					
Intercept for Middle	-0.5907	0.0451	-13.1026	<.0001	-0.6791	-0.5023
log(GDPpc) for Middle	0.0605	0.0054	11.1771	<.0001	0.0499	0.0711
R^2 for Middle	43.24%					
Intercept for Richer	-0.4106	0.0317	-12.9634	<.0001	-0.4727	-0.3486
log(GDPpc) for Richer	0.0437	0.0038	11.4054	<.0001	0.0362	0.0512
R^2 for Richer	46.05%					

Table R: Effect of Wealth Quintiles on Obesity, across GDP per capita

	Estimate	SE	Z-value	p-value	Lower CI	Upper CI
Intercept for Poorest	-0.2013	0.0387	-5.2048	<.0001	-0.2771	-0.1255
log(GDPpc) for Poorest	0.0159	0.0046	3.4352	0.0006	0.0068	0.0250
R^2 for Poorest	5.47%					
Intercept for Poorer	-0.2468	0.0320	-7.7225	<.0001	-0.3094	-0.1841
log(GDPpc) for Poorer	0.0232	0.0038	6.0253	<.0001	0.0156	0.0307
R^2 for Poorer	16.83%					
Intercept for Middle	-0.2430	0.0266	-9.1391	<.0001	-0.2951	-0.1909
log(GDPpc) for Middle	0.0243	0.0032	7.5690	<.0001	0.0180	0.0306
R^2 for Middle	25.77%					
Intercept for Richer	-0.1936	0.0195	-9.9176	<.0001	-0.2319	-0.1553
log(GDPpc) for Richer	0.0206	0.0024	8.6447	<.0001	0.0159	0.0252
R^2 for Richer	34.73%					

Table S: Effect of Wealth Quintiles on BMI, across GDP per capita

	Estimate	SE	Z-value	p-value	Lower CI	Upper CI
Intercept for Poorest	-7.0088	0.7554	-9.2788	<.0001	-8.4893	-5.5284
log(GDPpc) for Poorest	0.6431	0.0901	7.1343	<.0001	0.4664	0.8198
R^2 for Poorest	22.89%					
Intercept for Poorer	-7.2780	0.6261	-11.6240	<.0001	-8.5052	-6.0508
log(GDPpc) for Poorer	0.7188	0.0748	9.6078	<.0001	0.5721	0.8654
R^2 for Poorer	35.73%					
Intercept for Middle	-6.3942	0.5040	-12.6864	<.0001	-7.3821	-5.4064
log(GDPpc) for Middle	0.6517	0.0603	10.8007	<.0001	0.5335	0.7700
R^2 for Middle	41.33%					
Intercept for Richer	-4.2432	0.3495	-12.1392	<.0001	-4.9283	-3.5581
log(GDPpc) for Richer	0.4455	0.0421	10.5872	<.0001	0.3631	0.5280
R^2 for Richer	42.08%					

8 Forecasting and Out-of-Sample Validation

Our forecasting methodology uses a fixed effects regression to project overweight and obesity prevalence rates by age, sex, and personal wealth decile to 2040. First, we estimate the fixed effects model using all available survey data (ranging from 1995 to 2016). We store the coefficient estimates and the associated variance-covariance matrix. Second, we use the Institute for Health Metrics and Evaluation's GDP per capita series (specifically, the 1,000 draws described in the Data section) to estimate 1,000 versions of country-age-sex-wealth specific obesity prevalence rates. Additionally, to propagate model uncertainty, we use the coefficient means and variance-covariance matrix to draw parameter estimates to be used for each of the 1,000 forecast draws. Finally, we use the World Population Prospects to aggregate our estimates to the country-year-wealth level.

8.1 Forecasted Prevalence of Overweight

In the Table T, we report the estimated prevalence of overweight by country, wealth quintile, and year (2000, 2020, and 2040).

Table T: Appendix Projections by Country (2000, 2020, 2040)

ISO3	Quintile	Mean (2000)	Up (2000)	Low (2000)	Mean (2020)	Up (2020)	Low (2020)	Mean (2040)	Up (2040)	Low (2040)
ALB	1	24	24	24	38	38	38	58	59	58
ALB	2	29	29	29	44	44	44	63	64	63
ALB	3	33	33	33	47	47	47	65	67	64
ALB	4	38	38	38	50	50	49	66	69	64
ALB	5	44	44	44	48	50	47	61	67	57
ARE	1	51	51	51	70	71	70	84	84	83
ARE	2	55	55	55	74	74	74	86	87	86
ARE	3	55	55	55	75	75	74	86	87	85
ARE	4	50	50	50	72	73	71	85	86	82
ARE	5	34	34	34	59	62	57	75	81	69
ARM	1	29	29	29	44	45	44	63	63	62
ARM	2	34	34	34	50	51	50	68	68	67
ARM	3	39	39	39	54	54	54	70	72	69
ARM	4	47	47	47	58	59	57	72	75	70
ARM	5	57	57	57	59	61	57	71	76	66
AUT	1	33	33	33	51	51	51	68	68	68
AUT	2	38	38	38	56	56	55	72	72	72
AUT	3	39	39	39	57	57	56	73	73	73
AUT	4	38	38	38	55	55	54	71	72	70
AUT	5	30	30	30	43	44	43	60	62	58
AZE	1	35	35	35	52	52	52	69	69	69
AZE	2	41	41	41	57	58	57	73	74	73
AZE	3	46	46	46	60	61	59	75	76	74
AZE	4	53	53	53	61	63	60	76	78	74
AZE	5	61	61	61	58	60	55	72	77	67
BDI	1	3	3	3	6	6	6	12	13	12
BDI	2	4	4	4	8	8	8	16	16	16
BDI	3	6	6	6	11	11	11	21	22	20
BDI	4	9	9	9	17	18	17	31	33	29
BDI	5	20	20	20	32	34	31	51	56	46
BEL	1	34	34	34	51	51	51	68	68	68
BEL	2	39	39	39	56	56	56	72	72	72
BEL	3	40	40	40	57	57	57	73	73	72
BEL	4	39	39	39	55	56	55	71	72	70
BEL	5	31	31	31	45	45	44	61	63	58

Table T: Appendix Projections by Country (2000, 2020, 2040)

ISO3	Quintile	Mean (2000)	Up (2000)	Low (2000)	Mean (2020)	Up (2020)	Low (2020)	Mean (2040)	Up (2040)	Low (2040)
BEN	1	9	9	9	17	17	17	30	30	30
BEN	2	11	11	11	21	21	21	36	36	35
BEN	3	14	14	14	25	25	25	41	42	40
BEN	4	20	20	20	33	33	32	48	50	47
BEN	5	31	31	31	45	46	44	58	61	54
BFA	1	2	2	2	5	5	5	10	10	10
BFA	2	3	3	3	7	7	7	13	13	12
BFA	3	4	4	4	8	8	8	15	16	15
BFA	4	7	7	7	12	12	12	19	21	18
BFA	5	13	13	13	19	20	18	25	29	21
BGD	1	5	5	5	10	10	10	20	20	20
BGD	2	7	7	7	13	13	13	24	24	24
BGD	3	8	8	8	15	15	15	27	28	26
BGD	4	12	12	12	19	19	19	30	32	28
BGD	5	20	20	20	24	25	24	30	35	27
BIH	1	24	24	24	40	40	40	58	59	58
BIH	2	29	29	29	46	46	45	64	64	63
BIH	3	33	33	33	49	49	49	66	67	65
BIH	4	38	38	38	52	53	51	68	70	66
BIH	5	42	42	42	51	53	50	65	71	60
BOL	1	35	35	35	53	53	53	71	71	70
BOL	2	41	41	41	59	59	59	75	76	75
BOL	3	46	46	46	63	63	63	78	78	77
BOL	4	53	53	53	67	67	67	80	81	79
BOL	5	60	60	60	70	70	69	80	82	77
BRA	1	21	21	21	36	36	36	55	55	55
BRA	2	25	25	25	41	41	41	60	61	60
BRA	3	27	27	27	44	44	44	63	64	62
BRA	4	30	30	30	46	47	45	65	67	63
BRA	5	30	30	30	44	46	42	62	66	58
CIV	1	13	13	13	23	23	23	38	39	38
CIV	2	16	16	16	28	28	28	45	45	44
CIV	3	19	19	19	32	32	32	49	50	47
CIV	4	25	25	25	38	39	37	55	58	51
CIV	5	34	34	34	46	47	44	60	66	53

Table T: Appendix Projections by Country (2000, 2020, 2040)

ISO3	Quintile	Mean (2000)	Up (2000)	Low (2000)	Mean (2020)	Up (2020)	Low (2020)	Mean (2040)	Up (2040)	Low (2040)
CMR	1	16	16	16	29	29	29	46	46	46
CMR	2	20	20	20	34	35	34	52	53	52
CMR	3	24	24	24	39	40	39	57	58	56
CMR	4	31	31	31	47	47	46	62	64	61
CMR	5	42	42	42	56	57	55	68	72	64
COD	1	4	4	4	7	7	7	14	14	14
COD	2	5	5	5	9	9	9	18	19	18
COD	3	7	7	7	12	12	12	23	24	22
COD	4	12	12	12	19	19	18	33	36	30
COD	5	25	25	25	33	35	31	51	57	44
COG	1	13	13	13	24	24	24	40	41	39
COG	2	16	16	16	29	29	29	46	47	45
COG	3	19	19	19	33	33	32	51	52	49
COG	4	24	24	24	38	39	37	57	60	53
COG	5	30	30	30	43	46	41	63	69	56
COL	1	29	29	29	46	46	46	65	65	65
COL	2	34	34	34	52	52	51	70	70	69
COL	3	38	38	38	54	55	54	72	73	71
COL	4	42	42	42	57	57	56	73	74	72
COL	5	44	44	44	55	56	54	70	73	68
COM	1	17	17	17	30	30	30	48	48	48
COM	2	21	21	21	37	37	36	55	56	55
COM	3	26	26	26	43	43	42	61	62	61
COM	4	34	34	34	53	53	53	71	72	69
COM	5	49	49	49	68	69	67	82	84	80
CZE	1	32	32	32	50	50	50	66	66	66
CZE	2	36	36	36	55	56	55	70	71	70
CZE	3	39	39	39	57	57	57	71	72	70
CZE	4	39	39	39	56	57	56	69	71	68
CZE	5	35	35	35	47	48	47	58	62	55
DEU	1	31	31	31	48	48	47	66	66	65
DEU	2	36	36	36	52	52	52	70	70	69
DEU	3	37	37	37	53	54	53	70	71	70
DEU	4	36	36	36	51	52	51	68	69	67
DEU	5	28	28	28	40	41	40	56	58	54

Table T: Appendix Projections by Country (2000, 2020, 2040)

ISO3	Quintile	Mean (2000)	Up (2000)	Low (2000)	Mean (2020)	Up (2020)	Low (2020)	Mean (2040)	Up (2040)	Low (2040)
DNK	1	29	29	29	45	45	45	64	64	63
DNK	2	33	33	33	50	50	50	68	68	68
DNK	3	35	35	35	51	51	51	69	69	68
DNK	4	33	33	33	49	49	49	67	68	66
DNK	5	25	25	25	38	39	37	55	57	53
DOM	1	30	30	30	46	47	46	65	65	64
DOM	2	35	35	35	52	52	52	69	70	69
DOM	3	38	38	38	55	55	54	71	72	70
DOM	4	43	43	43	57	57	56	70	72	68
DOM	5	45	45	45	54	55	52	63	68	58
ECU	1	23	23	23	39	39	39	58	58	58
ECU	2	28	28	28	45	45	45	63	64	63
ECU	3	32	32	32	48	48	48	66	67	66
ECU	4	36	36	36	51	52	51	69	70	68
ECU	5	40	40	40	52	53	50	68	71	65
EGY	1	68	68	68	82	82	82	90	90	90
EGY	2	73	73	73	85	85	85	92	92	92
EGY	3	76	76	76	86	87	86	93	93	93
EGY	4	79	79	79	88	88	88	93	94	93
EGY	5	81	81	81	87	88	87	92	93	91
ESP	1	31	31	31	50	50	50	66	66	65
ESP	2	35	35	35	55	55	55	70	70	70
ESP	3	37	37	37	56	56	56	71	71	70
ESP	4	37	37	37	55	56	55	69	70	67
ESP	5	30	30	30	46	47	45	58	61	54
EST	1	28	28	28	44	45	44	61	61	60
EST	2	32	32	32	50	50	49	66	66	65
EST	3	35	35	35	51	52	51	67	68	66
EST	4	37	37	37	51	52	50	65	68	63
EST	5	35	35	35	43	45	42	56	61	50
ETH	1	2	2	2	5	5	5	10	10	9
ETH	2	3	3	3	6	6	6	12	13	12
ETH	3	5	5	5	8	8	8	14	15	13
ETH	4	8	8	8	11	11	10	18	20	15
ETH	5	17	17	17	17	18	16	21	28	16

Table T: Appendix Projections by Country (2000, 2020, 2040)

ISO3	Quintile	Mean (2000)	Up (2000)	Low (2000)	Mean (2020)	Up (2020)	Low (2020)	Mean (2040)	Up (2040)	Low (2040)
FIN	1	30	30	30	45	45	45	63	64	63
FIN	2	34	34	34	50	50	50	68	68	67
FIN	3	35	35	35	52	52	51	69	70	68
FIN	4	35	35	35	50	51	49	67	69	65
FIN	5	27	27	27	40	41	39	56	60	53
FRA	1	28	28	28	44	44	44	62	62	62
FRA	2	32	32	32	49	49	49	66	67	66
FRA	3	34	34	34	50	50	50	67	68	67
FRA	4	33	33	33	49	49	49	66	67	65
FRA	5	26	26	26	39	40	38	55	58	53
GAB	1	25	25	25	41	41	41	60	61	60
GAB	2	29	29	29	47	47	47	65	66	65
GAB	3	32	32	32	49	50	49	68	69	67
GAB	4	34	34	34	51	52	50	69	71	67
GAB	5	31	31	31	48	50	46	66	70	61
GBR	1	28	28	28	45	45	45	62	63	62
GBR	2	33	33	33	50	50	50	67	67	67
GBR	3	34	34	34	51	51	51	68	68	67
GBR	4	34	34	34	50	50	49	66	67	65
GBR	5	26	26	26	40	40	39	55	57	53
GEO	1	21	21	21	35	35	35	52	52	51
GEO	2	26	26	26	40	41	40	57	58	56
GEO	3	30	30	30	44	44	43	60	61	59
GEO	4	37	37	37	47	48	46	62	64	59
GEO	5	46	46	46	48	50	46	60	65	54
GHA	1	13	13	13	24	24	24	39	40	39
GHA	2	17	17	17	29	29	29	45	46	44
GHA	3	20	20	20	33	33	33	49	50	48
GHA	4	26	26	26	39	39	38	53	55	51
GHA	5	36	36	36	45	47	44	55	60	50
GIN	1	8	8	8	14	14	14	26	26	26
GIN	2	10	10	10	18	18	18	32	32	31
GIN	3	13	13	13	22	22	22	37	37	36
GIN	4	18	18	18	30	30	29	45	46	42
GIN	5	29	29	29	43	44	42	55	59	51

Table T: Appendix Projections by Country (2000, 2020, 2040)

ISO3	Quintile	Mean (2000)	Up (2000)	Low (2000)	Mean (2020)	Up (2020)	Low (2020)	Mean (2040)	Up (2040)	Low (2040)
GRC	1	30	30	30	49	49	48	66	66	66
GRC	2	35	35	35	54	54	54	71	71	70
GRC	3	37	37	37	56	56	56	72	73	71
GRC	4	37	37	37	56	57	55	72	74	71
GRC	5	31	31	31	49	51	48	66	69	63
GTM	1	27	27	27	44	44	44	63	63	63
GTM	2	32	32	32	50	50	50	68	69	68
GTM	3	36	36	36	54	54	53	71	72	71
GTM	4	41	41	41	58	58	58	74	75	73
GTM	5	46	46	46	61	61	60	75	77	73
GUY	1	31	31	31	47	47	47	67	67	66
GUY	2	37	37	37	53	53	52	72	73	71
GUY	3	42	42	42	57	57	56	74	76	73
GUY	4	49	49	49	61	62	60	77	79	75
GUY	5	56	56	56	64	66	62	76	81	72
HND	1	32	32	32	50	50	50	69	70	69
HND	2	38	38	38	56	56	56	74	75	74
HND	3	43	43	43	61	61	60	77	78	77
HND	4	50	50	50	66	67	66	81	82	80
HND	5	59	59	59	71	72	71	83	85	81
HRV	1	27	27	27	43	43	43	61	61	61
HRV	2	32	32	32	48	49	48	66	66	65
HRV	3	34	34	34	51	51	50	67	68	66
HRV	4	36	36	36	51	52	50	67	69	65
HRV	5	34	34	34	45	47	44	59	64	55
HTI	1	9	9	9	17	17	17	32	32	31
HTI	2	11	11	11	22	22	21	38	38	37
HTI	3	14	14	14	26	26	26	44	45	43
HTI	4	19	19	19	35	35	34	54	56	51
HTI	5	30	30	30	48	50	47	67	71	63
HUN	1	30	30	30	48	48	48	65	65	65
HUN	2	35	35	35	53	53	53	69	70	69
HUN	3	38	38	38	55	55	55	70	71	70
HUN	4	39	39	39	55	55	55	69	70	68
HUN	5	36	36	36	48	49	47	59	62	57
IND	1	6	6	6	11	12	11	22	22	21
IND	2	8	8	8	14	14	14	26	26	25
IND	3	10	10	10	16	17	16	28	29	27
IND	4	13	13	13	19	19	19	29	31	27
IND	5	20	20	20	21	22	20	25	30	22

Table T: Appendix Projections by Country (2000, 2020, 2040)

ISO3	Quintile	Mean (2000)	Up (2000)	Low (2000)	Mean (2020)	Up (2020)	Low (2020)	Mean (2040)	Up (2040)	Low (2040)
IRL	1	35	35	35	54	55	54	70	70	69
IRL	2	40	40	40	59	59	59	73	74	73
IRL	3	42	42	42	59	60	59	73	74	72
IRL	4	40	40	40	56	57	56	68	70	66
IRL	5	32	32	32	42	44	41	49	55	45
ISR	1	28	28	28	45	45	45	62	62	62
ISR	2	32	32	32	50	50	50	67	67	66
ISR	3	34	34	34	51	51	51	68	68	67
ISR	4	34	34	34	50	51	50	66	68	65
ISR	5	28	28	28	41	42	41	56	59	54
ITA	1	29	29	29	46	46	46	64	64	63
ITA	2	33	33	33	51	51	51	68	68	68
ITA	3	34	34	34	53	53	53	69	70	69
ITA	4	34	34	34	52	52	52	68	69	67
ITA	5	26	26	26	43	43	42	58	61	56
JOR	1	51	51	51	70	70	70	83	83	83
JOR	2	57	57	57	74	75	74	86	86	86
JOR	3	61	61	61	77	77	77	88	88	87
JOR	4	66	66	66	79	80	79	89	90	88
JOR	5	70	70	70	80	81	78	88	90	86
KAZ	1	26	26	26	42	42	42	58	59	58
KAZ	2	31	31	31	47	47	47	63	64	63
KAZ	3	34	34	34	49	50	49	65	66	64
KAZ	4	38	38	38	49	50	48	64	66	61
KAZ	5	40	40	40	43	45	41	55	60	50
KEN	1	9	9	9	18	18	18	31	32	31
KEN	2	12	12	12	22	22	22	37	37	36
KEN	3	14	14	14	26	26	26	41	43	41
KEN	4	20	20	20	32	32	31	48	50	46
KEN	5	29	29	29	41	42	39	54	59	50
KGZ	1	20	20	20	34	34	34	51	51	51
KGZ	2	24	24	24	40	40	40	57	58	57
KGZ	3	29	29	29	45	45	44	62	63	61
KGZ	4	37	37	37	52	53	51	68	70	66
KGZ	5	49	49	49	61	62	59	75	77	71
KHM	1	5	5	5	9	9	9	18	19	18
KHM	2	6	6	6	12	12	12	22	23	22
KHM	3	8	8	8	14	14	14	25	26	24
KHM	4	12	12	12	18	18	18	28	31	26
KHM	5	21	21	21	23	24	22	29	35	25

Table T: Appendix Projections by Country (2000, 2020, 2040)

ISO3	Quintile	Mean (2000)	Up (2000)	Low (2000)	Mean (2020)	Up (2020)	Low (2020)	Mean (2040)	Up (2040)	Low (2040)
LAO	1	6	6	6	11	11	11	21	21	21
LAO	2	7	7	7	13	13	13	25	25	24
LAO	3	9	9	9	15	16	15	27	28	26
LAO	4	12	12	12	18	18	18	27	30	26
LAO	5	19	19	19	20	21	19	24	29	21
LBR	1	7	7	7	14	14	13	25	26	25
LBR	2	9	9	9	18	18	17	32	32	31
LBR	3	12	12	12	22	23	22	39	40	37
LBR	4	19	19	19	32	33	31	51	54	48
LBR	5	35	35	35	51	54	49	71	76	65
LKA	1	10	10	10	18	18	18	31	31	30
LKA	2	12	12	12	21	22	21	35	36	35
LKA	3	14	14	14	24	24	23	37	38	36
LKA	4	17	17	17	26	26	25	37	39	35
LKA	5	21	21	21	24	26	23	30	35	27
LSO	1	24	24	24	39	39	39	58	58	57
LSO	2	29	29	29	45	45	45	64	64	63
LSO	3	34	34	34	50	51	50	68	68	67
LSO	4	44	44	44	58	58	57	72	74	71
LSO	5	57	57	57	66	67	65	76	79	73
LUX	1	36	36	36	53	53	53	70	70	69
LUX	2	40	40	40	57	58	57	73	74	73
LUX	3	41	41	41	57	58	57	73	74	72
LUX	4	37	37	37	53	53	52	68	70	66
LUX	5	25	25	25	36	37	35	50	55	46
LVA	1	27	27	27	44	44	44	61	61	60
LVA	2	32	32	32	49	49	49	65	66	65
LVA	3	35	35	35	51	52	51	67	68	66
LVA	4	38	38	38	51	52	51	66	68	64
LVA	5	38	38	38	45	47	43	57	62	53
MAR	1	26	26	26	43	44	43	61	62	61
MAR	2	32	32	32	49	50	49	67	67	66
MAR	3	36	36	36	53	54	53	69	70	68
MAR	4	42	42	42	57	58	56	71	73	69
MAR	5	49	49	49	59	61	58	70	74	65
MDA	1	26	26	26	42	42	42	61	62	61
MDA	2	32	32	32	48	49	48	67	67	66
MDA	3	37	37	37	53	53	52	70	71	69
MDA	4	46	46	46	59	59	58	74	76	72
MDA	5	58	58	58	64	66	62	77	81	72

Table T: Appendix Projections by Country (2000, 2020, 2040)

ISO3	Quintile	Mean (2000)	Up (2000)	Low (2000)	Mean (2020)	Up (2020)	Low (2020)	Mean (2040)	Up (2040)	Low (2040)
MDG	1	3	3	3	6	6	6	13	13	13
MDG	2	4	4	4	8	8	8	16	16	16
MDG	3	5	5	5	11	11	10	20	20	19
MDG	4	8	8	8	15	16	15	27	28	25
MDG	5	15	15	15	25	26	25	39	42	35
MDV	1	27	27	27	46	46	46	65	65	64
MDV	2	33	33	33	51	52	51	69	70	69
MDV	3	36	36	36	54	54	54	71	72	71
MDV	4	40	40	40	56	57	55	72	74	70
MDV	5	43	43	43	53	54	52	67	71	64
MEX	1	25	25	25	41	42	41	61	61	60
MEX	2	29	29	29	47	47	47	66	66	65
MEX	3	32	32	32	50	50	49	68	68	67
MEX	4	34	34	34	51	52	51	68	70	67
MEX	5	32	32	32	47	48	47	64	67	61
MLI	1	8	8	8	15	15	15	27	27	27
MLI	2	10	10	10	19	19	19	32	33	32
MLI	3	13	13	13	23	24	23	37	39	36
MLI	4	19	19	19	31	31	30	45	48	42
MLI	5	31	31	31	43	44	41	54	60	49
MMR	1	7	7	7	13	13	13	24	25	24
MMR	2	9	9	9	16	17	16	29	29	28
MMR	3	12	12	12	19	19	18	31	32	29
MMR	4	17	17	17	22	23	21	32	35	29
MMR	5	28	28	28	24	26	23	28	34	23
MOZ	1	6	6	6	10	10	10	20	20	20
MOZ	2	8	8	8	14	14	13	25	25	24
MOZ	3	10	10	10	17	17	17	30	31	29
MOZ	4	16	16	16	24	25	24	38	41	36
MOZ	5	32	32	32	38	40	37	51	57	46
MRT	1	17	17	17	30	30	30	48	48	47
MRT	2	21	21	21	36	36	36	54	55	53
MRT	3	26	26	26	41	41	40	59	60	58
MRT	4	32	32	32	47	48	46	65	68	62
MRT	5	43	43	43	55	57	53	71	76	66
MUS	1	23	23	23	38	38	38	57	57	57
MUS	2	27	27	27	43	43	43	62	62	61
MUS	3	30	30	30	45	45	45	63	64	62
MUS	4	32	32	32	46	46	45	62	63	60
MUS	5	32	32	32	41	42	40	52	56	48

Table T: Appendix Projections by Country (2000, 2020, 2040)

ISO3	Quintile	Mean (2000)	Up (2000)	Low (2000)	Mean (2020)	Up (2020)	Low (2020)	Mean (2040)	Up (2040)	Low (2040)
MWI	1	7	7	7	13	13	13	25	26	25
MWI	2	9	9	9	17	17	17	31	32	31
MWI	3	12	12	12	22	22	21	37	38	36
MWI	4	18	18	18	31	31	30	48	50	46
MWI	5	33	33	33	47	49	46	65	69	60
MYS	1	21	21	21	35	35	35	54	54	54
MYS	2	25	25	25	40	40	40	59	59	59
MYS	3	27	27	27	42	42	42	60	61	59
MYS	4	29	29	29	42	42	41	59	60	57
MYS	5	27	27	27	35	36	34	48	52	45
NAM	1	17	17	17	30	30	30	48	48	47
NAM	2	21	21	21	35	35	35	53	54	53
NAM	3	24	24	24	38	38	38	56	57	56
NAM	4	28	28	28	41	42	40	59	61	57
NAM	5	31	31	31	41	42	40	58	62	54
NER	1	7	7	7	13	13	13	23	23	23
NER	2	9	9	9	16	17	16	29	29	28
NER	3	12	12	12	21	21	21	34	36	33
NER	4	19	19	19	30	30	29	45	47	42
NER	5	35	35	35	47	49	45	61	66	56
NGA	1	11	11	11	20	20	20	34	35	34
NGA	2	14	14	14	24	24	24	40	41	39
NGA	3	17	17	17	27	28	27	44	46	43
NGA	4	23	23	23	32	33	31	50	53	47
NGA	5	33	33	33	37	39	34	55	61	49
NLD	1	31	31	31	47	47	47	65	65	65
NLD	2	36	36	36	52	52	52	69	70	69
NLD	3	37	37	37	53	53	53	70	70	69
NLD	4	36	36	36	51	51	50	67	68	66
NLD	5	27	27	27	39	40	39	54	57	52
NOR	1	30	30	30	47	47	47	64	65	64
NOR	2	34	34	34	51	51	51	69	69	68
NOR	3	35	35	35	52	52	52	69	69	68
NOR	4	33	33	33	49	49	48	66	67	65
NOR	5	23	23	23	35	36	35	52	54	50
NPL	1	5	5	5	10	10	10	21	21	21
NPL	2	7	7	7	13	13	13	26	26	25
NPL	3	9	9	9	16	16	16	30	30	29
NPL	4	13	13	13	21	21	21	35	37	34
NPL	5	22	22	22	29	30	28	42	46	38

Table T: Appendix Projections by Country (2000, 2020, 2040)

ISO3	Quintile	Mean (2000)	Up (2000)	Low (2000)	Mean (2020)	Up (2020)	Low (2020)	Mean (2040)	Up (2040)	Low (2040)
PAK	1	17	17	17	29	29	29	47	47	46
PAK	2	21	21	21	35	35	35	53	53	52
PAK	3	24	24	24	39	39	39	56	57	56
PAK	4	30	30	30	45	45	44	60	62	59
PAK	5	39	39	39	50	51	49	61	65	59
PER	1	36	36	36	54	54	54	71	72	71
PER	2	42	42	42	60	60	59	76	76	75
PER	3	46	46	46	63	63	62	77	78	76
PER	4	51	51	51	65	66	64	78	80	76
PER	5	56	56	56	64	66	62	75	79	71
PHL	1	9	9	9	16	16	16	29	30	29
PHL	2	11	11	11	20	20	20	34	35	34
PHL	3	13	13	13	23	23	22	37	38	36
PHL	4	16	16	16	26	26	25	38	41	36
PHL	5	21	21	21	27	28	26	35	40	31
PRT	1	29	29	29	48	48	47	65	65	65
PRT	2	34	34	34	53	53	53	69	70	69
PRT	3	36	36	36	55	55	54	71	71	70
PRT	4	36	36	36	54	55	54	70	71	68
PRT	5	31	31	31	47	48	46	60	64	57
PRY	1	19	19	19	32	32	32	51	51	50
PRY	2	23	23	23	37	37	37	56	57	56
PRY	3	26	26	26	41	41	40	59	60	58
PRY	4	31	31	31	44	45	44	61	62	59
PRY	5	36	36	36	45	46	44	58	62	54
RUS	1	29	29	29	45	46	45	62	62	61
RUS	2	34	34	34	51	51	51	67	67	66
RUS	3	36	36	36	53	53	52	68	69	67
RUS	4	39	39	39	53	54	52	68	70	66
RUS	5	38	38	38	47	48	45	62	66	57
RWA	1	7	7	7	13	13	13	24	25	24
RWA	2	9	9	9	17	17	17	29	30	29
RWA	3	12	12	12	20	21	20	33	35	32
RWA	4	18	18	18	27	28	26	39	43	36
RWA	5	33	33	33	38	40	36	46	53	39
SEN	1	11	11	11	20	20	20	34	35	34
SEN	2	14	14	14	25	25	25	40	41	39
SEN	3	17	17	17	29	30	29	45	46	43
SEN	4	23	23	23	37	37	36	51	53	49
SEN	5	34	34	34	47	49	46	58	62	53

Table T: Appendix Projections by Country (2000, 2020, 2040)

ISO3	Quintile	Mean (2000)	Up (2000)	Low (2000)	Mean (2020)	Up (2020)	Low (2020)	Mean (2040)	Up (2040)	Low (2040)
SLE	1	10	10	10	19	19	18	33	33	33
SLE	2	13	13	13	23	23	23	40	40	39
SLE	3	17	17	17	28	29	28	46	47	45
SLE	4	25	25	25	37	38	36	56	58	54
SLE	5	41	41	41	52	54	50	69	73	65
STP	1	18	18	18	31	31	31	49	49	48
STP	2	22	22	22	37	37	37	55	56	54
STP	3	26	26	26	42	42	42	59	60	58
STP	4	34	34	34	49	50	48	65	67	62
STP	5	47	47	47	58	60	56	70	74	65
SVK	1	29	29	29	47	47	46	63	64	63
SVK	2	34	34	34	52	52	52	68	68	67
SVK	3	36	36	36	53	54	53	69	69	68
SVK	4	38	38	38	53	53	52	67	68	65
SVK	5	36	36	36	45	45	44	56	60	52
SVN	1	29	29	29	46	46	46	62	62	62
SVN	2	33	33	33	51	51	51	66	67	66
SVN	3	35	35	35	53	53	53	67	68	67
SVN	4	36	36	36	52	53	52	66	67	64
SVN	5	31	31	31	44	45	43	55	59	51
SWE	1	28	28	28	44	44	44	61	62	61
SWE	2	32	32	32	49	49	48	66	66	65
SWE	3	34	34	34	50	50	49	66	67	66
SWE	4	33	33	33	48	48	47	64	65	62
SWE	5	25	25	25	37	37	36	51	55	47
SWZ	1	39	39	39	56	56	56	74	75	74
SWZ	2	44	44	44	62	62	62	79	79	78
SWZ	3	49	49	49	66	66	65	81	81	80
SWZ	4	54	54	54	69	70	69	83	84	82
SWZ	5	57	57	57	71	72	70	83	86	81
TCD	1	8	8	8	15	15	14	27	27	27
TCD	2	10	10	10	18	19	18	33	34	32
TCD	3	13	13	13	22	23	22	38	40	37
TCD	4	20	20	20	30	31	29	47	51	44
TCD	5	33	33	33	42	44	39	60	66	53
TGO	1	11	11	11	20	21	20	35	36	35
TGO	2	14	14	14	25	26	25	41	42	41
TGO	3	17	17	17	31	31	30	47	49	46
TGO	4	25	25	25	40	41	39	56	59	53
TGO	5	39	39	39	55	57	53	68	73	62

Table T: Appendix Projections by Country (2000, 2020, 2040)

ISO3	Quintile	Mean (2000)	Up (2000)	Low (2000)	Mean (2020)	Up (2020)	Low (2020)	Mean (2040)	Up (2040)	Low (2040)
TJK	1	14	14	14	26	26	25	42	42	42
TJK	2	18	18	18	31	31	31	48	49	48
TJK	3	23	23	23	36	36	35	53	54	52
TJK	4	31	31	31	43	44	43	60	62	58
TJK	5	46	46	46	53	55	52	68	72	64
TLS	1	3	3	3	5	5	5	11	11	10
TLS	2	4	4	4	7	7	7	13	14	13
TLS	3	5	5	5	8	9	8	16	17	15
TLS	4	7	7	7	11	12	11	21	23	19
TLS	5	12	12	12	17	18	15	28	32	24
TUN	1	19	19	19	34	34	34	52	52	52
TUN	2	23	23	23	40	40	40	58	58	57
TUN	3	26	26	26	43	43	43	61	61	60
TUN	4	30	30	30	46	46	46	63	64	62
TUN	5	32	32	32	46	47	45	62	65	60
TUR	1	50	50	50	68	68	67	81	82	81
TUR	2	55	55	55	72	72	72	84	85	84
TUR	3	58	58	58	74	74	73	85	86	84
TUR	4	61	61	61	74	75	73	84	86	82
TUR	5	60	60	60	69	70	67	77	81	72
TZA	1	8	8	8	16	16	16	28	28	27
TZA	2	11	11	11	20	20	20	33	34	32
TZA	3	14	14	14	23	24	23	37	38	36
TZA	4	20	20	20	30	30	29	42	45	40
TZA	5	32	32	32	38	40	37	47	53	42
UGA	1	9	9	9	17	17	16	30	30	29
UGA	2	11	11	11	21	21	21	36	36	35
UGA	3	15	15	15	25	25	25	41	42	40
UGA	4	21	21	21	32	33	32	49	51	47
UGA	5	35	35	35	44	46	43	59	63	56
UKR	1	28	28	28	45	45	45	62	62	61
UKR	2	33	33	33	51	51	50	67	68	66
UKR	3	37	37	37	54	55	54	70	71	69
UKR	4	43	43	43	58	59	57	74	76	72
UKR	5	49	49	49	60	62	58	76	79	72
URY	1	25	25	25	41	41	41	60	60	59
URY	2	30	30	30	46	47	46	64	65	64
URY	3	33	33	33	49	49	48	66	67	65
URY	4	35	35	35	49	50	48	65	67	63
URY	5	35	35	35	44	45	42	57	62	52

Table T: Appendix Projections by Country (2000, 2020, 2040)

ISO3	Quintile	Mean (2000)	Up (2000)	Low (2000)	Mean (2020)	Up (2020)	Low (2020)	Mean (2040)	Up (2040)	Low (2040)
VNM	1	6	6	6	12	12	12	21	22	21
VNM	2	7	7	7	14	15	14	25	26	25
VNM	3	9	9	9	17	17	17	27	28	27
VNM	4	13	13	13	19	20	19	29	31	27
VNM	5	19	19	19	22	22	21	26	30	23
ZAF	1	34	34	34	52	52	52	70	71	70
ZAF	2	40	40	40	58	58	58	75	75	75
ZAF	3	43	43	43	61	61	61	77	78	77
ZAF	4	47	47	47	64	64	63	79	80	78
ZAF	5	48	48	48	63	64	62	78	81	76
ZMB	1	10	10	10	19	20	19	34	34	33
ZMB	2	13	13	13	24	24	24	40	40	39
ZMB	3	17	17	17	28	28	27	44	45	43
ZMB	4	23	23	23	34	34	33	50	53	48
ZMB	5	34	34	34	41	42	40	56	62	51
ZWE	1	14	14	14	27	27	27	45	45	44
ZWE	2	18	18	18	33	33	33	52	53	51
ZWE	3	22	22	22	39	39	38	58	59	56
ZWE	4	28	28	28	48	49	47	67	69	64
ZWE	5	37	37	37	61	63	59	77	82	72

To understand the performance of the mean estimate, we randomly withheld survey-years and estimated those country-years using a regression based on the remaining data. Specifically we withheld 10 countries randomly from our sample (about 10% of the sample). This means that the estimation will not include information from any time points observed from that country (be it 1 year of observation or 6 years of observation). We did this because we wish to see how accurately GDP per capita predicts the wealth-obesity gradient shift without information about that country. We estimated the projections for those withheld countries and compared the estimates to what was actually observed (and withheld). The vast majority of our data is from low and middle income countries, so this table displays information mostly about those countries. We see that the model under-estimated the wealth-obesity gradient shift in these countries. The results presented below display the average relative error of those predictions to the data in the held-out country-years in the obesity forecast.

Table U: Out-of-Sample Cross Validation for Obesity Prevalence

Personal Wealth Decile	Relative Error	Predicted Fraction	Actual Fraction
1	.6031641	.0679341	.0405114
2	.6533905	.0734711	.0475292
3	.5727391	.0868214	.0495829
4	.6191857	.0920583	.0568844
5	.6342006	.0999395	.0632354
6	.8046303	.1046064	.0842451
7	.9070861	.1137974	.1032994
8	1.173965	.1171718	.1374058
9	1.485211	.1227564	.1816984
10	1.953695	.1214436	.2356081
